# Reactive oxygen species-scavenging nanomaterials for the prevention and treatment of age-related diseases

**DOI:** 10.1186/s12951-024-02501-9

**Published:** 2024-05-15

**Authors:** Yun Dai, Yifan Guo, Weicheng Tang, Dan Chen, Liru Xue, Ying Chen, Yican Guo, Simin Wei, Meng Wu, Jun Dai, Shixuan Wang

**Affiliations:** 1 Department of Obstetrics and Gynecology, Tongji Hospital, Tongji Medical College, Huazhong University of Science and Technology, Wuhan, 430030 Hubei China; 2 National Clinical Research Center for Obstetrical and Gynecological Diseases, Wuhan, 430030 Hubei China; 3 Key Laboratory of Cancer Invasion and Metastasis, Ministry of Education, Wuhan, 430030 Hubei China; 4 Department of Marine Pharmacy, College of Food and Pharmaceutical Sciences, Ningbo University, Ningbo, 315800 China

**Keywords:** Aging, Age-related disease, Nanomaterials, Antioxidant, Reactive oxygen species

## Abstract

With increasing proportion of the elderly in the population, age-related diseases (ARD) lead to a considerable healthcare burden to society. Prevention and treatment of ARD can decrease the negative impact of aging and the burden of disease. The aging rate is closely associated with the production of high levels of reactive oxygen species (ROS). ROS-mediated oxidative stress in aging triggers aging-related changes through lipid peroxidation, protein oxidation, and DNA oxidation. Antioxidants can control autoxidation by scavenging free radicals or inhibiting their formation, thereby reducing oxidative stress. Benefiting from significant advances in nanotechnology, a large number of nanomaterials with ROS-scavenging capabilities have been developed. ROS-scavenging nanomaterials can be divided into two categories: nanomaterials as carriers for delivering ROS-scavenging drugs, and nanomaterials themselves with ROS-scavenging activity. This study summarizes the current advances in ROS-scavenging nanomaterials for prevention and treatment of ARD, highlights the potential mechanisms of the nanomaterials used and discusses the challenges and prospects for their applications.

**Graphical Abstract:**

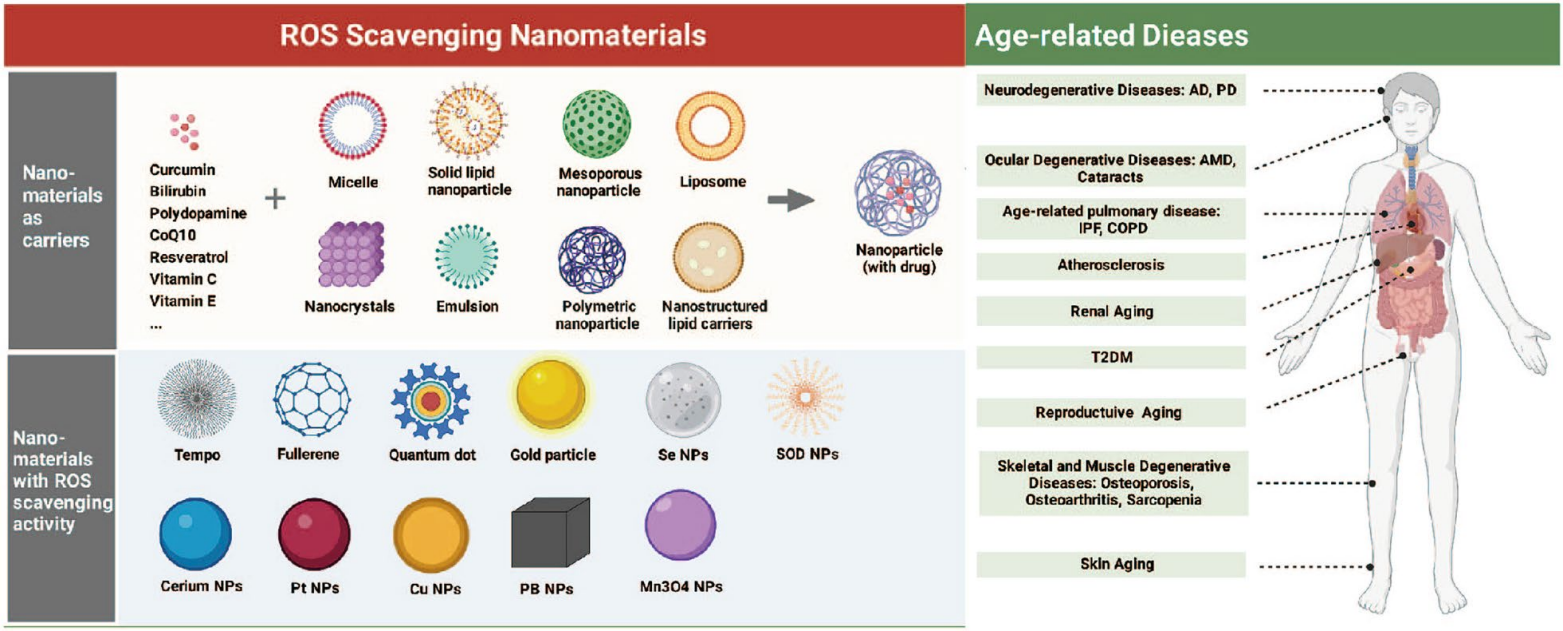

## Introduction

With the ameliorating sanitary conditions and the continuous development of the economic status, population aging becomes a major global phenomenon revealing a steady increase in life expectancy among geriatrics. According to the World Population Prospects 2019 issued by United Nations, the global population of people over 65 years is expected to increase from 703 million to 1.5 billion by 2050 (from approximately 9% in 2019 to nearly 16% in 2050) [1]. Aging has become a major health concern in the world as it is the primary driver for most chronic diseases, such as cardiovascular diseases, type 2 diabetes, glaucoma, obesity, Alzheimer’s disease, Parkinson’s disease, macular degeneration, and osteoarthritis. Given the increasing aging population and age-related adverse side effects, it is crucial to understand the molecular mechanisms underlying aging and explore more efficient therapeutic strategies [2, 3].

Scientists have been exploring mechanisms of aging and developing methods to postpone senility, including cross-linkage theory of aging [4], the free radical theory [5], telomere shortening theory [6], and immune senescence theory of aging [7]. The free radical theory of aging posits that excessive reactive oxygen species (ROS) and oxidative stress (OS) cause oxidative damage and abnormal functioning of biomolecules (e.g. DNA, proteins and lipids), leading to damage of cells and tissues. In this process, organisms gradually lose their functional and adaptive capacity to the point of aging [5, 8, 9]. ROS are a class of free radicals, including oxidizing substances such as superoxide anion (·O_2_·), hydrogen peroxide (H_2_O_2_), hydroxyl radical (OH-), and singlet oxygen (^1^O_2_). OH- are one of the most reactive ROS. OH- and ·O_2_· can react with other molecules, leading to lipid peroxidation, protein oxidation, and DNA damage, thus triggering cell damage and death. In addition, ·O_2_· is a precursor to other ROS and can generate more toxic ROS species. Excessive amounts of H_2_O_2_ can lead to cytochrome oxidation, protein oxidation and DNA damage, ultimately leading to cell death. ^1^O_2_ can react with double-bond-containing biomolecules in organisms to cause oxidative damage. For example, it can trigger lipid peroxidation, which damages the integrity of cell membranes and affects cell function. Consequently, scavenging ROS by antioxidants may be an effective strategy for slowing the progression of aging-related disease. Antioxidants could approximately be classified as hydrophilic antioxidants like vitamin C, glutathione (GSH) and lipophilic antioxidants like vitamin E, carotene, coenzyme Q10 (CoQ10). Recent studies have revealed the pharmacological properties of antioxidants both in vivo and in vitro, while clinical trials involving antioxidants have produced largely disappointing results [10]. Antioxidants’ medicinal potential is constrained by their poor stability and low utilization [10]. Therefore, developing new methods for the application of antioxidants is essential.

ROS-Scavenging nanotechnology has emerged as an exciting and promising new means of treating age-related disease. Nanomaterials (NMs) are particles between 1 and 100 nm in size. Due to their nanoscale size, these particles have greater surface area and higher surface-to-volume ratios, have higher mechanical strength, and are quite stable [11]. NMs can be utilized as medication carriers. In order to preserve tiny molecules from degradation or to facilitate the absorption and distribution of natural antioxidants, polymeric nanoparticles are used to encapsulate or integrate the molecules. Additionally, NMs offer weak water-soluble antioxidants greater solubility and improved surface functionalization to produce target-specificity. Some nanoparticles (NPs) that have a quenching impact on ROS can be used directly as antioxidants. Fullerene (C60) and its derivatives and other inorganic NMs with inherent catalytic characteristics (such as platinum (Pt) and gold (Au)) are examples of common ROS-detoxifying nanoplatforms [12–14]. ROS Scavenging nanotechnology show great potential in the prevention and treatment of ARD.

In this perspective, we investigate the use of ROS-scavenging nanotechnology in ARD, discussing its safety, prospective uses, potential applications, and translational challenges in order to promote progress in the development of new treatments.

## Reactive oxygen species and the oxidative stress theory of aging

### Source of ROS

The free radical theory of aging is predicated on the premise that age-related functional declines are the result of ROS-induced damage accumulation. ROS are a group of oxygen-containing chemical substances that are highly reactive, mainly generated by redox reactions in the organism. ROS are classified as either free radicals or non-free radicals [15]. Free-radical ROS includes superoxide anion radical (·O_2_·), hydroxyl radical (OH), peroxyl radical (ROO), and sulfhydryl peroxyl radical (RSOO). Non-free radical ROS includes hydrogen peroxide (H_2_O_2_), organic hydroperoxides (ROOH), ozone (O_3_) and singlet oxygen (^1^O_2_). Excessive ROS can cause the disruption of the balance between the pro-oxidant and anti-oxidants, leading to OS [16].

**Fig. 1:**
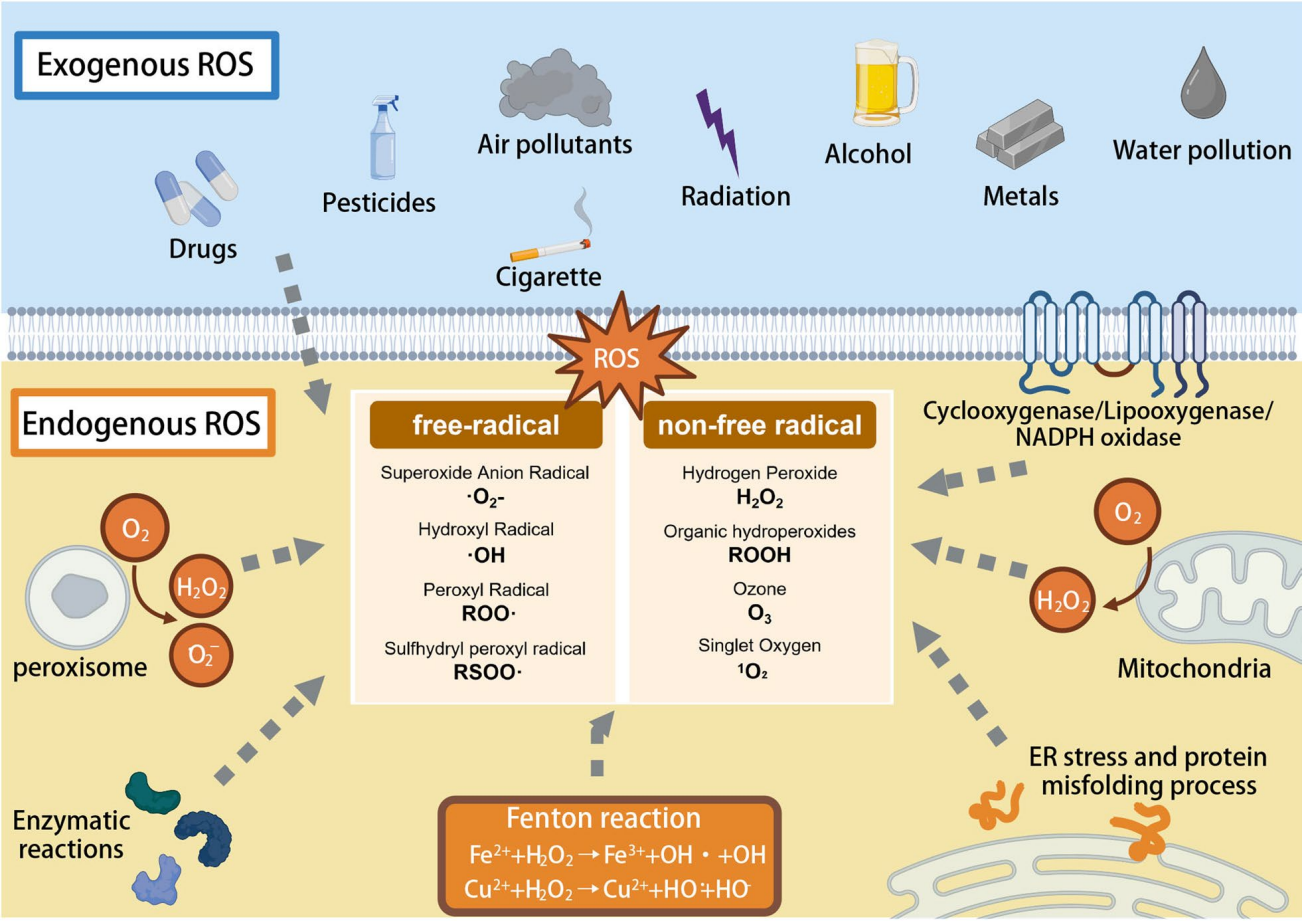
Categories and source of ROS. Created with BioRender.com

ROS are generated in multiple compartments and by a variety of enzymes within the cell, and there are endogenous and exogenous ROS in body [17] (Fig. 1). Endogenous ROS are mainly produced directly by various organelles such as mitochondria, cytoplasmic membrane, endoplasmic reticulum (ER), peroxisomes, and lysosomes. The most significant source of ROS production occurs mainly in the mitochondrial electron transport chain (ETC) complexes I, II and III, due to electron leakage [18, 19]. ETC transfers electrons from NADH to O_2_ and generate ·O_2_·, which can then be rapidly broken down to H_2_O_2_ by superoxide dismutase (SOD). When Fe^2+^ and Cu^2+^ are present, H_2_O_2_ can also be converted to ·OH through the Fenton reaction. The protein misfolding process that occurs in ER is also accompanied by the production of ROS [19]. ROS production on ER is generated by delivering electrons to O_2_ by NADH-cytochrome P450 reductase to form ·O_2_·, with electrons delivered to O_2_ by the electron transport chain on the nuclear membrane, assisted by NADH [20, 21]. In addition, various types of oxidase such as NADPH oxidase (NOX), cytochrome P450 (CYP) enzymes, xanthine oxidase (XO), nitric oxide synthase (NOS), which promote the production of endogenous ROS [22, 23]. Hypoxanthine can be converted to xanthine catalyzed by XO in a process accompanied by the reduction of O_2_ to ·O_2_·. Endothelial nitric oxide synthase (eNOS) produce ·O_2_·. Monoamine oxidase, lipoxygenase and cyclooxygenase enzymes, can also promote the production of ROS in normal biological reactions. Additionally, genetic factors can potentially contribute to oxidative stress. The copper (zinc) superoxide dismutase 1 (SOD-1) gene is the most prevalent genetic contributor to amyotropic lateral sclerosis (ALS), which accounts for 5–10% of cases. The mutations in this gene, which enhance oxidative stress in the cells, promote protein deposition, disrupt intracellular calcium ions, and cause the diffusion of toxicity, are responsible for around 20% of familial ALS and 2% of sporadic ALS [24]. Exogenous ROS are induced by external factors including alcohol, cigarette smoke, heavy metals (lead, chromium), industrial solvents, pesticides, medications like halothane and nonsteroidal anti-inflammatory medicines, radiation, and other pollutants such as air and water pollutants [25]. In addition, ischemia–reperfusion (I/R) damage, infections, and inflammation all lead to increased levels of ROS [26].

### Oxidative damage of ROS in aging

**Fig. 2:**
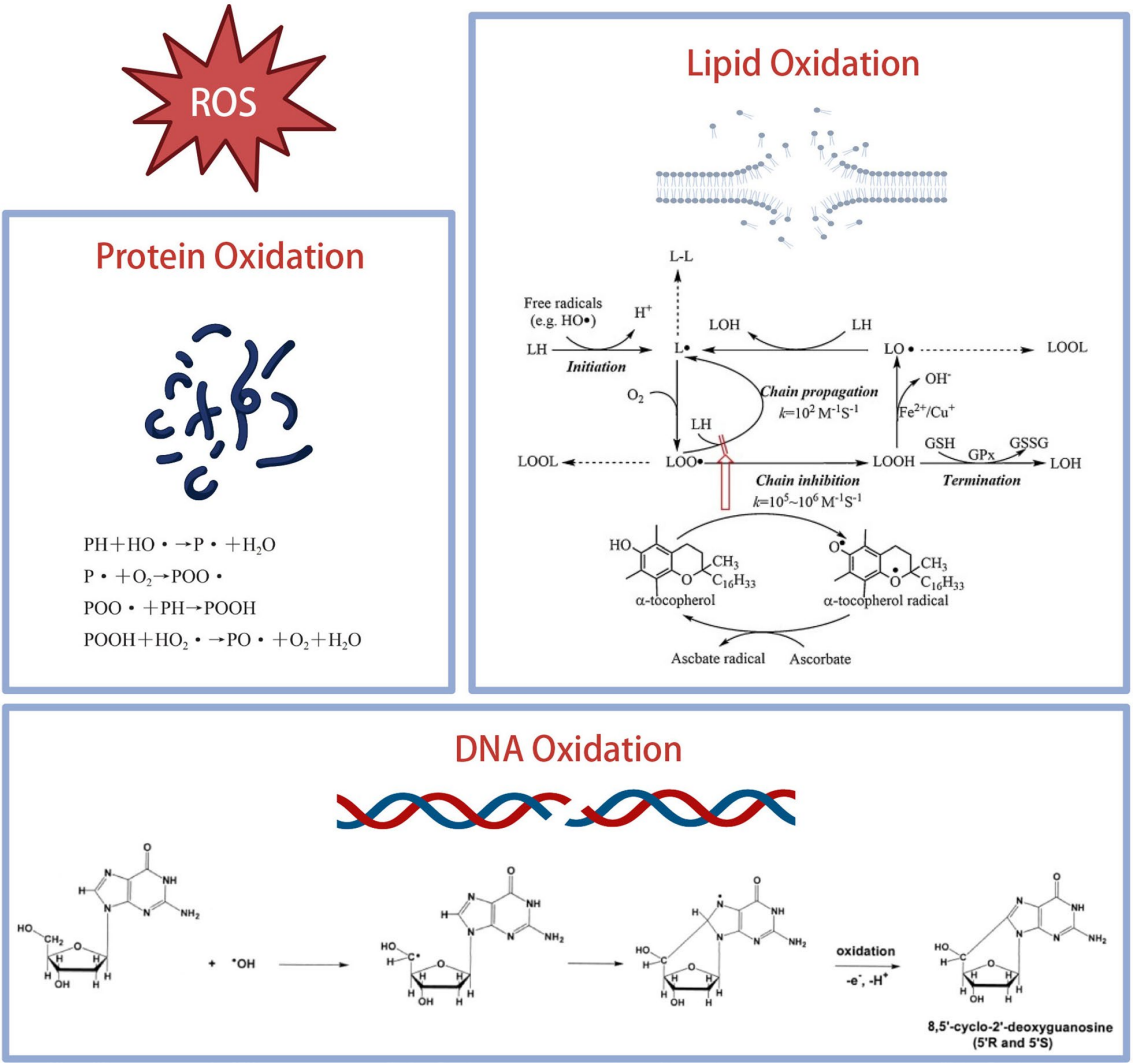
Oxidative damage of protein, lipid and DNA. Copyright 2020, Elsevier. Copyright 2002, Elsevier

The aging process is a loss of internal homeostasis due to the accumulation of molecular damage to macromolecules such as DNA, lipids and proteins. Under physiological circumstances, the intracellular generation and scavenging of ROS is usually in homeostasis [27]. At low concentrations, ROS participates in cell growth and survival, immune response, metabolic regulation, and cell signaling process [28, 29]. OS is determined by an imbalance between ROS generation and antioxidant defenses, which gradually damages biomolecules including DNA, lipids, and proteins by oxidation [30] (Fig. 2). Harman formulated the free radical theory of aging, indicating that free-radical associated macromolecular damage may promote senescence [5].

#### Lipid peroxidation

Polyunsaturated fatty acid (PUFA), especially PUFA with more double bonds, such as arachidonic acid and linoleic acid, are highly susceptible to ROS and free radicals [31]. Since PUFA is the main component of cell membranes, cell membranes are vulnerable to free radical damage; when membrane phospholipids come into contact with an inordinate amount of ROS, lipid peroxidation occurs. This extensive lipid peroxidation alters the membrane's structure, reducing its fluidity and compromising its integrity [32]. Moreover, Lipid peroxides are also extremely reactive substances that have the ability to generate more ROS or breakdown into reactive substances that can crosslink proteins and DNA. They interact with free amino groups in proteins, causing them to covalently modify, cross-link, oligomerize, and aggregate. These mechanisms, which produce intracellular damage, decrease cell activities and induce cell death, have been linked to aging and a variety of ARD.

#### Protein oxidation

Exposure of proteins to ROS results in multiple changes, including amino acid residues oxidation, protein fragmentation due to oxidative cleavage of the peptide backbone, irreversible production of protein carbonyl compounds and generation of protein–protein cross-linkages [33–36]. With the accumulation of oxidative damage, proteins are more likely to misfold. Moderately oxidized proteins are degraded by the proteasome [37], while severely oxidized proteins can cross-link with other proteins, thus preventing their degradation [38]. As a result, severely damaged proteins accumulate within the cell, altering physiological properties such as loss of catalytic activity and paralysis of regulation of metabolic pathways. It is known that dysfunctions in the cellular apparatus of protein quality control contribute to aging and ARD, such as neurodegenerative and cardiovascular diseases [39].

#### DNA oxidation

ROS generate major OS when they react with nitrogenous bases and deoxyribose. DNA oxidation damage mainly include base mutation, strand breaking, DNA–protein cross-links, and formation of DNA-adducts [33]. Direct strand excision and oxidative damage to pyrimidine and purine bases are both effects of hydroxyl radical stress on DNA. In addition to oxidizing DNA bases, ROS may potentially disrupt DNA strands by attacking the DNA backbone with free radicals [40, 41]. Furthermore, adducts to DNA can be formed through the reaction of deoxyguanosine and other macromolecular modifications triggered by ROS [42]. In addition, mitochondrial DNA (mtDNA) is highly susceptible to ROS, and has a significantly higher mutation rate than nuclear DNA. Histones and other chromatin-associated proteins present in the nuclear genome, which function as free radical scavengers, but not in the mitochondrial genome [43]. The persistence and accumulation of damaged mtDNA in the mitochondria inevitably lead to more ROS production, which in turn cause further damage. DNA damage can cause aging by affecting transcription, apoptosis signaling or cellular senescence or through somatic mutations and telomere shortening [44–46]. Continuous oxidative damage to mtDNA has been linked to aging, inflammation, carcinogenesis, and the development of malignancy [47]. The DNA damage response which consists of the activation of checkpoint pathways, cell cycle arrest and DNA repair, removes most of ROS-induced DNA damage [48].

8-hydroxyguanosine (8-OHG) is the oxidized base that occurs most frequently in RNA. Guanine is initially reacted with by the extremely reactive hydroxyl radical, which subsequently produces 8-OHG after losing an electron (e −) and proton (H +) [49]. The oxidized RNA is substantially intact, while its translation fidelity has been severely diminished. The oxidative alteration of RNA disrupts the translational process and impairs protein synthesis, causing cell degeneration or even cell death [50].

## ROS-scavenging nanotechnology and scavenging mechanisms

**Fig. 3:**
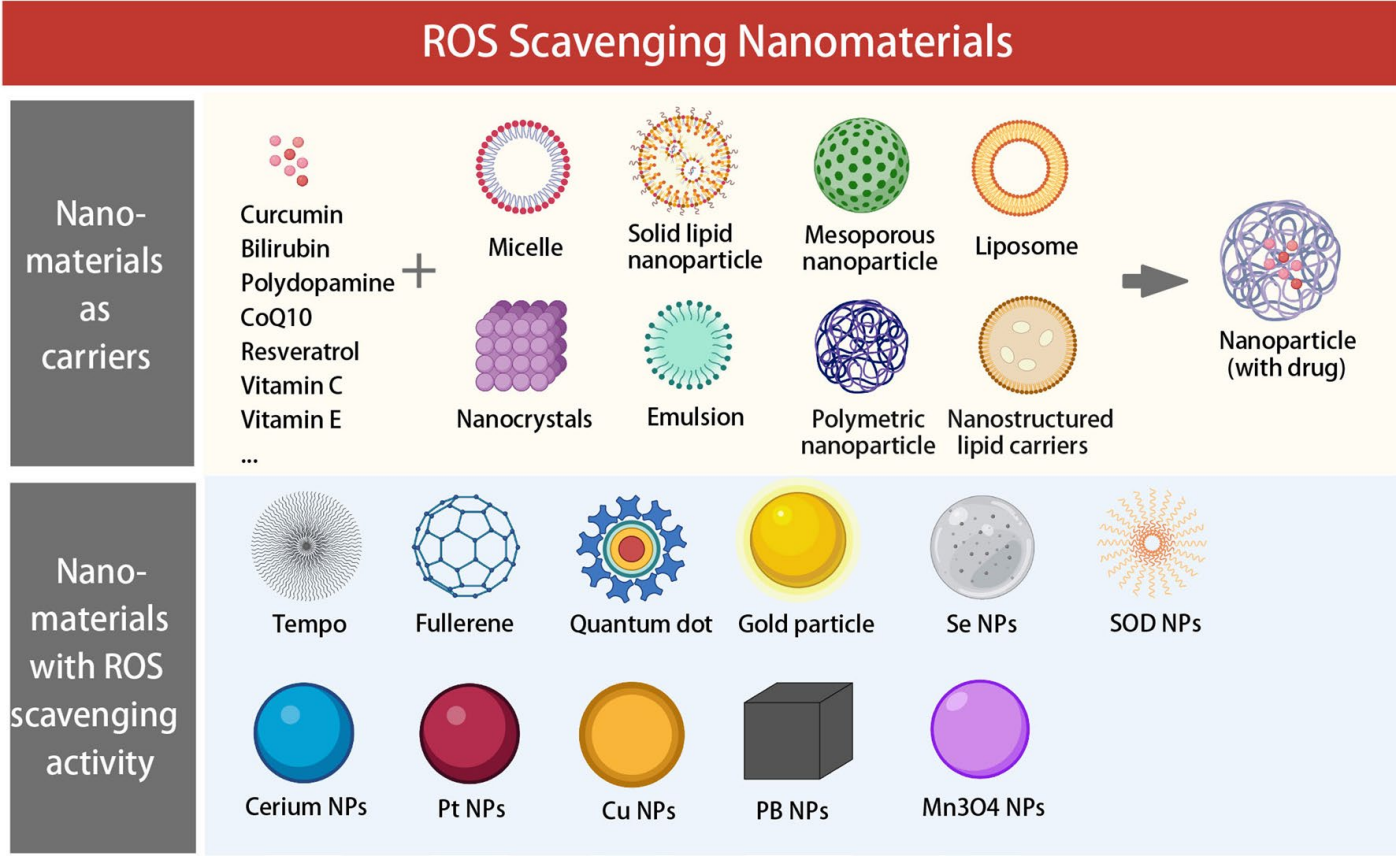
ROS-scavenging nanomaterials in treatment of ARD. Created with BioRender.com

A new window of opportunity has opened up for the advancement of conventional antioxidant therapy thanks to the recent proliferation of nanotechnology and nanoscience in the construction of ROS scavengers. It is possible to classify ROS-scavenging NMs as carriers for delivering natural antioxidants or nanomaterials with inherent ROS-scavenging activity (Fig. 3).

### Nanomaterials for catalytic generation of ROS-scavenging agents and its mechanisms

Nanomaterials with ROS scavenging activity include metals and metal oxides, carbon-based nanomaterials, enzyme-like nanoparticles, selenium and (2,2,6,6-tetramethylpiperidin-1-yl)oxyl (TEMPO). In the following section, we provide an overview of ROS-scavenging nanomaterials, focusing on their distinctive redox properties and mechanisms. Table 1 outlines the key aspects of select ROS scavengers.

**Table 1:** Nanomaterials with ROS scavenging activity and its mechanisms

Drug		mechanisms	References
Nanomaterials with ROS scavenging activity	CeO_2_ NPs	SOD- and CAT-mimetic nanozyme activities, convert ·O_2_· to O_2_, react with HO·, catalyze the degradation of H_2_O_2_, scavenging activity for ONOO −	[68–71]
	Pt NPs	POD-, CAT-, and SOD-like nanozyme activities, catalytically convert ·O_2_· to H_2_O_2_, and H_2_O_2_ to H_2_O and O_2_	[56]
	Cu NPs	POD-, CAT-, SOD-, and glutathione-like enzyme activities	[58]
	PB NPs	POD-, CAT-, and SOD-like multienzyme activities	[57]
	SOD-containing NPs	SOD enzyme activity, catalyze the neutralization of ·O_2_· to O_2_ and H_2_O_2_	[82]
	TEMPO	partially mimic SOD, capture ROS via the single electron on nitroxide	[81]
	Fullerene	SOD-like activity	[76]
	gold NPs	SOD,TAC-like activity	[83]
	Se NPs	Se is incorporated as selenocysteine (SEC) in various antioxidant enzymes like GPx, thioredoxin reductase (TXNRD) and selenoprotein P (SELENOP). Se acts as the redox centre of all these enzymes	[78, 79]
	Mn3O4 NPs	GPx, CAT, and SOD activity	[64]
Nanomaterials as carriers	Curcumin	Redox-activity due to low O–H bond dissociation energy	[84, 85]
	Bilirubin	Scavenge ·O_2_·, H_2_O_2_, and ·OH via an ROX-initiated redox reaction	[86]
	Polydopamine (PDA)	Scaveneg ·O_2_·, H_2_O_2_, and ·OH via redox chemistry of polycatechol structure	[87]
	CoQ10	shuttle electrons from complexes I and II to complex III of the mitochondrial respiratory chain	[88]
	resveratrol	maintain the expression of SOD1,CAT,GPx	[89, 90]
	Vitamin C	produce reactions with oxidizing agents through HAT, SET or a concerted transfer of electron/protons (SET/HAT), react with ·O_2_· and ·OH in the cytoplasm	[91]
	Vitamin E	prevent lipid peroxidation chain reactions and quenches O_2_ in cellular lipid compartments, reduce alkoxy radicals by transferring the phenolic hydrogen atom of the chroman ring reduces alkoxy radicals by transferring the phenolic hydrogen atom of the chroman ring	[92]
	H_2_	specifically neutralize OH and peroxynitrite, enhance the expression of heme oxygenase-1 (HO-1) by activating nuclear factor erythroid-related factor 2 (Nrf-2)	[93]

Strong catalytic activity is shown by nanoscale noble metal NMs like palladium (Pd), Au, and Pt, which is primarily ascribed to their huge specific surface area and larger fraction of metal atoms on their surfaces [51]. These nanocatalysts of noble metals have been proposed as possible antioxidants. Although AuNPs are not typically considered to possess redox activity, they serve as an ideal platform for electrochemical biosensors. This is because they can function as redox catalysts, thereby enhancing the electron transfer of various electroactive biological species (primarily redox proteins) without necessitating the use of electron transfer mediators [52]. Pt NMs are a viable choice to treat the oxidative damage due to their potent peroxidase POD-, CAT-, and SOD-like nanozyme activities that catalytically convert O_2_ to H_2_O_2_, H_2_O_2_ to H_2_O and O_2_ [53–56].

The high redox potential of Prussian blue (PB) NMs is due in large part to their high electron transfer capacity. Using an inflammatory model, Zhang et al. showed that PB NMs had the capacity to prevent or alleviate ROS-induced damage [57]. The antioxidant enzymes POD, CAT, and SOD are responsible for their catalytic activity and, by extension, their capacity to scavenge reactive oxygen species.

Copper (Cu) NMs possess excellent catalytic activity like POD-, CAT-, SOD-, and GSH-like enzyme activities [58]. It improves the body's capacity to rid itself of free radicals by increasing the efficiency with which SOD and other enzymes function [58–60].

Manganese (Mn) is an important element that plays a role in several cellular processes and metabolic reactions in the human body. The strong POD-, SOD-, and CAT-like activities of Mn^4+^ NMs have been shown in a number of different investigations [61–63]. Mn NMs (Mn^4+^) directly catalyze H_2_O_2_ to produce O_2_ and Mn^2+^. Then, Mn NMs (Mn^2+^) may imitate SOD function by reacting with ·O_2_· to produce H_2_O_2_. Mn_3_O_4_ NMs mimic the function of glutathione peroxidase (GPx), CAT, and SOD [64].

Due to the existence of Ce^3+^/Ce^4+^ (oxidized/reduced) and compensatory oxygen vacancies, cerium-based NMs have emerged as one of the most common ROS scavengers, enabling them to release or abstract an electron to neutralize different types of ROS [65–67]. In general, CeO_2_ NMs possess efficient redox activity to convert ·O_2_· to O_2_, react with HO·, catalyze the degradation of H_2_O_2_, scavenge ONOO − , exhibiting SOD (Ce^3+^) and CAT (Ce^4+^) mimetic activity to prevent oxidative injury to cells [68–71].

Nanomaterials having carbon frameworks, such as graphene, graphdiyne, and C60 and its derivatives, may be among the most prevalent ROS quenchers [72–74]. In the previous publication, the antioxidant capabilities of C60 and their derivatives were ascribed to the effectiveness of the C60 molecule, which can eliminate ROS through the C60's delocalized double bond system [75, 76]. C60 extinguishes ROS by accepting unpaired electrons, capable of receiving up to six electrons and accommodating as many as 34 methyl free radicals on the C60 sphere [75]. C60 has SOD-like activity [76].

Selenium (Se) functions as a redox center for GPx. Supplying with Se may raise GPx levels, increase H_2_O_2_ decomposition and decrease cell damage [77]. Selenoprotein P (SELENOP) and GPx are two of the antioxidant enzymes that assimilate Se NMs as selenocysteine (SEC). The redox center of these enzymes is the element Se [78, 79].

(2,2,6,6-tetramethylpiperidin-1-yl)oxyl (TEMPO) is a well-known ROS scavenger because it can capture unpaired electrons from other radicals by a single electron on nitroxide, and the redox reaction switch between oxidation states of nitroxide, oxoammonium cation, and hydroxylamine [80]. TEMPO is a membrane-permeable stable nitroxide radical that can scavenge superoxide and performs Fenton reactions and radical–radical recombination [81].

### Applications of nanocomposites in ROS-scavenging nanotechnology

A typical tactic for preserving redox equilibrium and minimizing OS damage is the introduction of extracellular ROS scavengers. Vitamin C, Vitamin E, CoQ10, resveratrol, MLT, quercetin, curcumin, H_2_ and other natural antioxidants make up the majority of the chemicals employed in the creation of antioxidant nanoparticles (Table 1). Nanomaterials can be used to composite not only natural antioxidants but also nano-enzymes to improve antioxidant properties and functionality. In addition to enhancing the stability and bioavailability of ROS scavenging drugs, NMs as delivery vehicles can also achieve targeted and controlled drug delivery. In the meantime, NMs as carriers may reduce the administered dose of medications, thereby minimizing adverse effects. By using a range of delivery vehicles, including liposomes, nanospheres, nanoemulsions and nanocrystals, the delivery techniques of the aforementioned non-enzymatic antioxidants have up till now been extensively explored (Table 2).

**Table 2:** Nanomaterials as carriers for delivering ROS scavenging drugs

Type	Definition	Feature	References
Liposomes	Closed vesicles formed by phospholipid or cholesterol bilayers, owe the hydrophilic and lipophilic properties	Liposomes improve penetration, boost solubilization, and serve as a local depot for prolonged release	[94]
		PEGylated phospholipids improve blood circulation time, drug loading and encapsulation efficiency, and stabilize drug encapsulation	[95]
Solid lipid nanocarriers (SLNs)	Consisting of lipids that are solid at both room temperature and body temperature	Control release of the drug and enhance physical stability	[96]
		Facilitate drug penetration through the epidermis and prolong drug release to prevent systemic absorption	[94]
		Increase stability, enhance drug load and reduce toxicity	[97]
		Increase drug stability, biocompatibility, increase protection of drugs against enzymatic metabolism and high drug loading capacity	[98]
		Show high stability against digestive enzymes	[99]
Nanostructured lipid-based nanocarriers (NLCs)	Consisting of lipids (both solid and liquid) distributed in surfactant-containing aqueous phases	Increase drug loading and stability	[96]
		Exhibit superior biocompatibility and protracted drug retention at the target site	[100]
Fe3O4@carbon dots	The core–shell nanoparticles with the magnetic nanoparticles	Provide excellent drug loading performance and fluorescence tracer function	[101]
Polymeric nanoparticles (PLGA)	Amphiphilic block copolymers self-assemble	Increase stability and in vivo persistence	[102]
		Exhibit tunable and sustained release and better stability	[103]
Nanoemulsions	Nanodroplets of one liquid are suspended in another liquid as part of a biphasic dispersion, which is stabilised by an amphiphilic surfactant	Improve the penetration and prolong the corneal retention time	[104]
		Maximum in vitro and ex vivo trans-nasal mucosal flux	[105]
Nanospheres	Particles with a diameter of 10–200 nm are used to dissolve, encapsulate, or link drugs to a polymer matrix	Serve as highly efficient delivery vehicles for drugs via transdermal and oral routes	[106]
Nanocrystals	Crystalline particles, produced by methods such as pearl milling, high pressure homogenization, precipitation, etc	Improve dissolution and pharmacokinetic behavior and similar photostability	[107]
Poly (D, L‐lactide co‐glycolide) (PLGA) NMs	Biodegradable and biocompatible copolymer consisting of lactic acid and glycolic acid	Efficient drug delivery, improved biosafety without overt adverse effects with prolonged therapy	[108]
		Prolong the release of nicotine	[109]
		Protect CAT from degradation in the biological milieu	[110]
		Improve stability of encapsulated medicines and controlled drug release	[111]
		Increase the biodistribution of phytol and keep it from leaking out of the brain and plasma	[112]
Poly (butyl cyanoacrylate) (PBCA) NMs	Composed of acrylic acid derivatives with low mammalian cytotoxicity	Improve the uptake, protect drugs from photodegradation and sustain drug release	[113]
Nanomicelles(phosphatidylethanolamine-distearoyl methoxypolyethylene glycol)	Consisting of amphiphilic blocks (hydrophobic and hydrophilic fragments) copolymers that form spherical copolymer micelles with an inner core–shell in water	Bioavailable, biodegradable, amphiphilic, biocompatible, nontoxic, extended circulators, and tiny enough to make in large quantities	[114]

## ROS-Scavenging nanotechnology in prevention and treatment of ARD

**Fig. 4:**
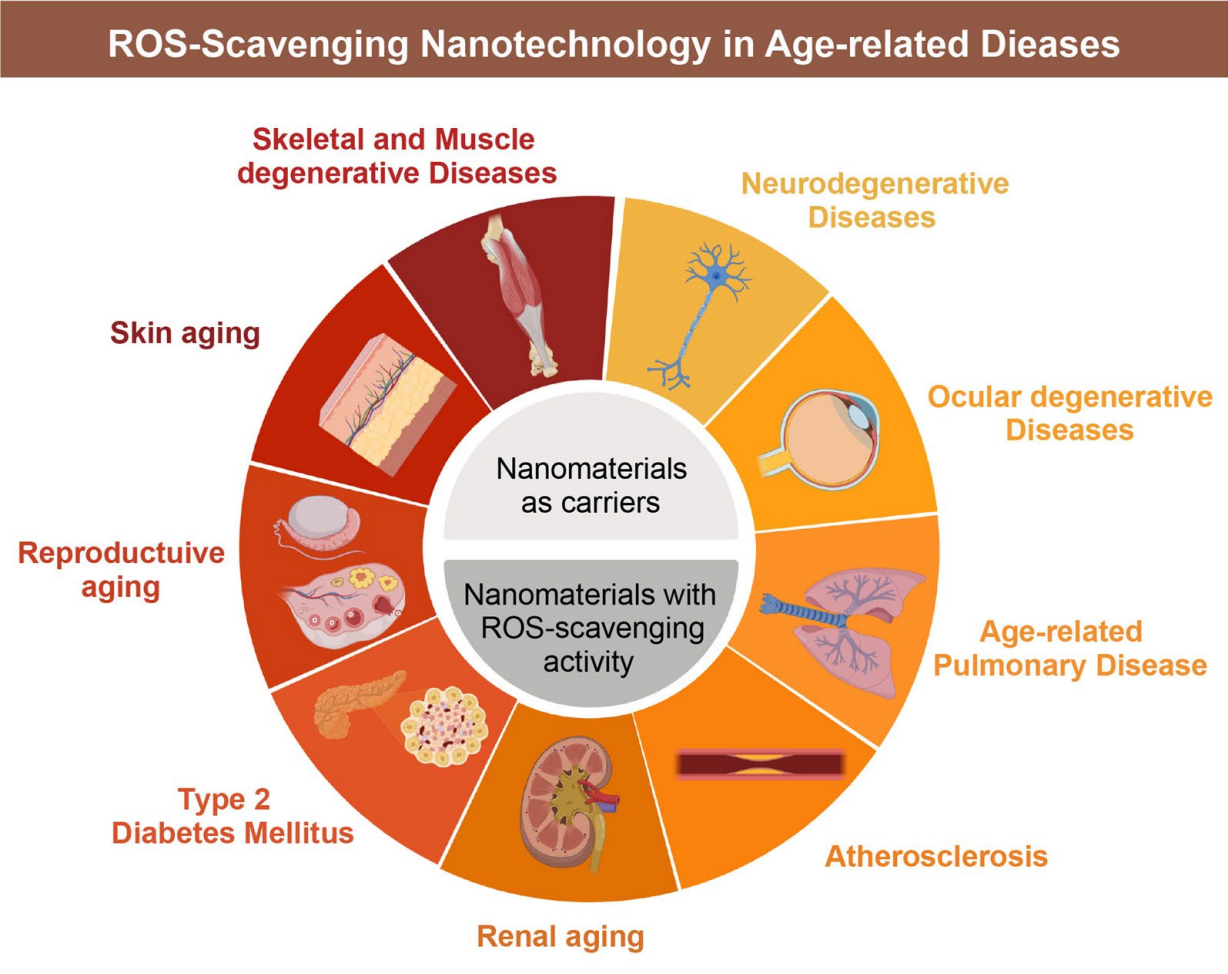
ROS-Scavenging nanotechnology in prevention and treatment of age-related diseases. Created with BioRender.com

Therapeutic interventions towards oxidative stress might allow restoring the health and curing the aged-related diseases that share basal processes. Overproduction of ROS leads to oxidative stress, which has been observed in diabetes, cardiovascular disease, idiopathic pulmonary fibrosis, neurodegenerative diseases, skeletal degenerative diseases, skin aging, reproductive system aging, and ocular aging. We focused on the implications of NPs-mediated ROS scavenger systems in aging and age-related diseases to provide insights into a potential intervention that may affect the aging process, and subsequently promote healthy longevity (Fig. 4).

### Type 2 diabetes mellitus

Type 2 diabetes mellitus (T2DM) is a burden on the geriatric population's health, afflicting almost 25% of those over 65 years old [115]. There are several recognized contributors to the pathophysiology of T2DM. ROS and OS play roles in all of them, including hyperglycemia, hyperlipidemia, inflammation, insulin resistance, and endothelial dysfunction. In hyperglycemic conditions, the polyol pathway attempts to reduce excess glucose to sorbitol by using NADPH. Thus NADPH is unable to produce the reduced GSH responsible for the inhibition of OS [116]. Under diabetic conditions, glucose is easily oxidized, causing the formation of H_2_O_2_ and other reactive byproducts [117]. There is evidence from clinical studies that strong correlations exist between the levels of pro-oxidants and OS-induced tissue damage indicators such as oxidation of DNA bases, 4-hydroxy-2-nonenal (HNE) proteins, hydroperoxides, 8-hydroxy-deoxyguanine, and 8-epi-prostaglandin [118–120]. Therefore, one of the greatest options to lessen the negative consequences of T2DM is antioxidant medication.

Se NMs have been utilized in conjunction with other nanomaterials to boost their antioxidant properties. Hanaa et al. treated diabetic mice with liposomes-Se (L-Se) nanoparticles. L-Se reduced serum glucose, pancreatic malondialdehyde (MDA), nitric oxide (NO), tumor necrosis factor-α (TNF-α), and prostaglandin F2α (PGF2α) levels. The treated diabetic mice also had higher serum insulin, pancreatic GSH, SOD, CAT, GPx, and GSH reductase (GR) levels [121]. Polysaccharide (RTFP-3)-functionalized Se nanoparticles (RP3-SeNPs) protected pancreatic islet cells in INS-1 cells from oxidative damage in another study. RTFP-3 owed high biocompatibility and biodegradability, while it exhibited antioxidant and α-glucosidase-inhibiting activities. RTFP-3 could generate synergistic effect with SeNPs [122]. The combination of Nanocerium and sodium selenite was verified that improved antioxidant enzymes and decreased oxidative stress more effectively than either alone [123].

Applications of AuNPs and ZnO NPs are being researched feverishly. AuNPs were discovered that they could inhibit lipid peroxidation and regulate antioxidant enzymes such as SOD, CAT, and GPx in diabetic mice. The AuNPs regulate hyperglycemia by scavenging free radicals, inhibiting the formation of ROS, and boosting antioxidant defense enzymes [83, 124]. Additionally, silver-gold nanoparticles (Ag@AuNP) with a core–shell structure were tested on diabetic rats. The Ag@AuNPs had better effects and lower expenses than AuNPs in reduction of blood glucose level and insulin resistance, as well as increasing insulin level [125].

ZnO NPs exhibit high antioxidant capabilities through the scavenging of ROS and the up-regulation of antioxidant enzyme activities. Furthermore, it had a hypoglycemic impact in diabetic mice via enhanced insulin production and glucose absorption by the liver, skeletal muscles, and adipose regions [126, 127]. Prissana et al. reported the treatment effects of doping silver (Ag) into the ZnO nanorods (ZnO:Ag NR's) in a diabetic murine model. The silver-doping strategy appears to effectively enhance the antioxidant potential of ZnO, as evidenced by their activities in scavenging NO, DPPH, and ·O_2_· [128].

Nanoparticles have limited use in diabetic treatment. Functionalized gadofullerene was later demonstrated to improve defective glycolipid metabolism in type 2 diabetic mice. However, gadofullerene's effect on clearance of ROS is negligible [129]. To ensure their success, it must be followed by carefully executed parallel bio-distribution and toxicity investigations.

### Atherosclerosis

The main pathological manifestation of Atherosclerosis (AS) is lipid deposition in some arterials with smooth muscle cells and fibrous matrix proliferation, which progressively develop into atherosclerotic plaques. There is a correlation between the degree of oxidation and the severity of AS. And it has been shown that oxidative changes of lipids and proteins have been found in vascular lesions [130]. Several processes involved in atherogenesis have been linked to ROS, including adhesion molecule expression, increased proliferation and migration of vascular smooth muscle, endothelial apoptosis, lipid peroxidation, matrix metalloproteinase activation and alterations in vasomotor activity [131]. Vascular endothelial cells experience chronic OS due to a decrease in the production of antioxidant enzymes such as SOD and CAT, leading to an increase in free radicals and ROS [132]. Hence, prevention of vascular OS represents crucial therapeutic strategy of AS.

Research on Nano-modification of traditional Chinese medicine is booming in AS, especially on the intelligent and biomimetic modification of their carriers.

Ginsenoside (Re) is a powerful component with anti-inflammatory and antioxidant characteristics [133, 134], as well as the ability to improve AS [135]. CAT and Re were co-loaded onto the surface of porous poly (lactic-coglycolic acid) (PLGA) NPs to develop a dual targeted model and multi-mechanism therapeutic biomimetic nanosystem (Cat/Re@PLGA@UCM) [108, 136]. The biomimetic nanosystem not only exhibit the ability to scavenge ROS, but also enable escaping macrophage phagocytosis and targeting atherosclerotic plaques, and H_2_O_2_-responsive drug release ability. The nanodrugs reduced atherosclerotic area 2.7-fold better than free Re.

Teng Wu et al. established a smart medication delivery device that adapted to the oxidative microenvironment of atherosclerotic plaques [137]. Poly (ethylene glycol) and poly (propylene sulphide) (PEG-PPS) was used to make andrographolide-loaded micelles. Andrographolide-loaded PEG-PPS micelles reduce inflammation and OS simultaneously. After oxidation, PPS becomes hydrophilic, improving medication distribution and effectiveness.

Meili et al. developed a smart system for reacting to the microenvironment of atherosclerotic plaques, which included ROS and shear stress. Red blood cells (RBCs) and simvastatin-loaded micelles (SV MC) comprised the system. RBCs were utilized to extend the circulation and improve the therapeutic effect. SV MC@RBCs micelles were ethylenediamine-functionalized ring-opened poly (glycidyl methacrylate)-poly (propylene sulfide) (PGED-PPS). The micelle ruptured when high ROS made hydrophobic PPS hydrophilic, releasing medication. PPS also reduces ROS, enabling synergistic AS therapy with medicines and materials [138].

Ferulic acid nanoparticles primarily inhibit the production of ROS by suppressing the expression of oxLDL receptors. Rebecca A. Chmielowski et al. developed ferulic acid-based poly (anhydride-ester) nanoparticles to reduce oxLDL absorption and ROS in human monocyte-derived macrophages (HMDMs) [139]. Ferulic acid-based polymer nanoparticles, which were biodegradable, may release ferulic acid sustainably and tunablely to inhibit macrophage foam cell production.

CeO_2_ nanoparticles could protect endothelial cells (ECs) from oxidative damage by counteracting H_2_O_2_-induced ROS [140]. Gao et al. found that the gadolinium doping of CeO_2_ (Gd/CeO_2_) nanozymes promoted the surface proportion of Ce^3+^ and ROS catalytic activity [141]. The optimized Gd/CeO_2_ nanozyme, which displayed optimal CAT and SOD mimic activities, revealed enhanced efficacy and anti-inflammatory benefits against AS via ROS salvage. Using probucol-loaded mesoporous polydopamine (MPDA) carriers and platelet membranes, Lu Chen et al. created a bionic multifunctional nanoplatform (BM-NP) [142]. BM-NPs selectively aggregated in plaque lesions of the ligated right carotid artery (RCA) animal model due to platelet membrane adherence to damaged blood arteries. BM-NPs' antioxidant properties may synergistically reduce plaque ROS and foamy macrophages, avoiding AS.

Metal NMs like MnO_2_, Au, and Pt have also been utilized in a wide range of researches. Mesoporous MnO_2_ nanoparticles with the modification of hyaluronic acid (HA) [143] reached high drug loading capacity of curcumin, which combined the catalytic activity of the nanocarrier and the antioxidant functions of curcumin. MnO_2_/HA showed intrinsic catalase mimic activity, which catalyzed the endogenous abundant H_2_O_2_ into O_2_ as self-oxygenation agent to relieve hypoxia in AS site. The resulting nanomedicine could also achieve targeting drug delivery by HA modification to bind CD44 receptor overexpressed on diseased macrophages surface.

Wang et al. produced raspberry-like Pt and cerium bimetallic nanostructures with ticagrelor loading and PEGylation (DPTP NRs) for synergistic AS treatment. Pt-cerium bimetallic nano-raspberry prevented foam cell formation by scavenging ROS and lowering plaque oxidized LDLs more effectively. Ticagrelor reduced plaque and platelet aggregation [144].

**Fig. 5:**
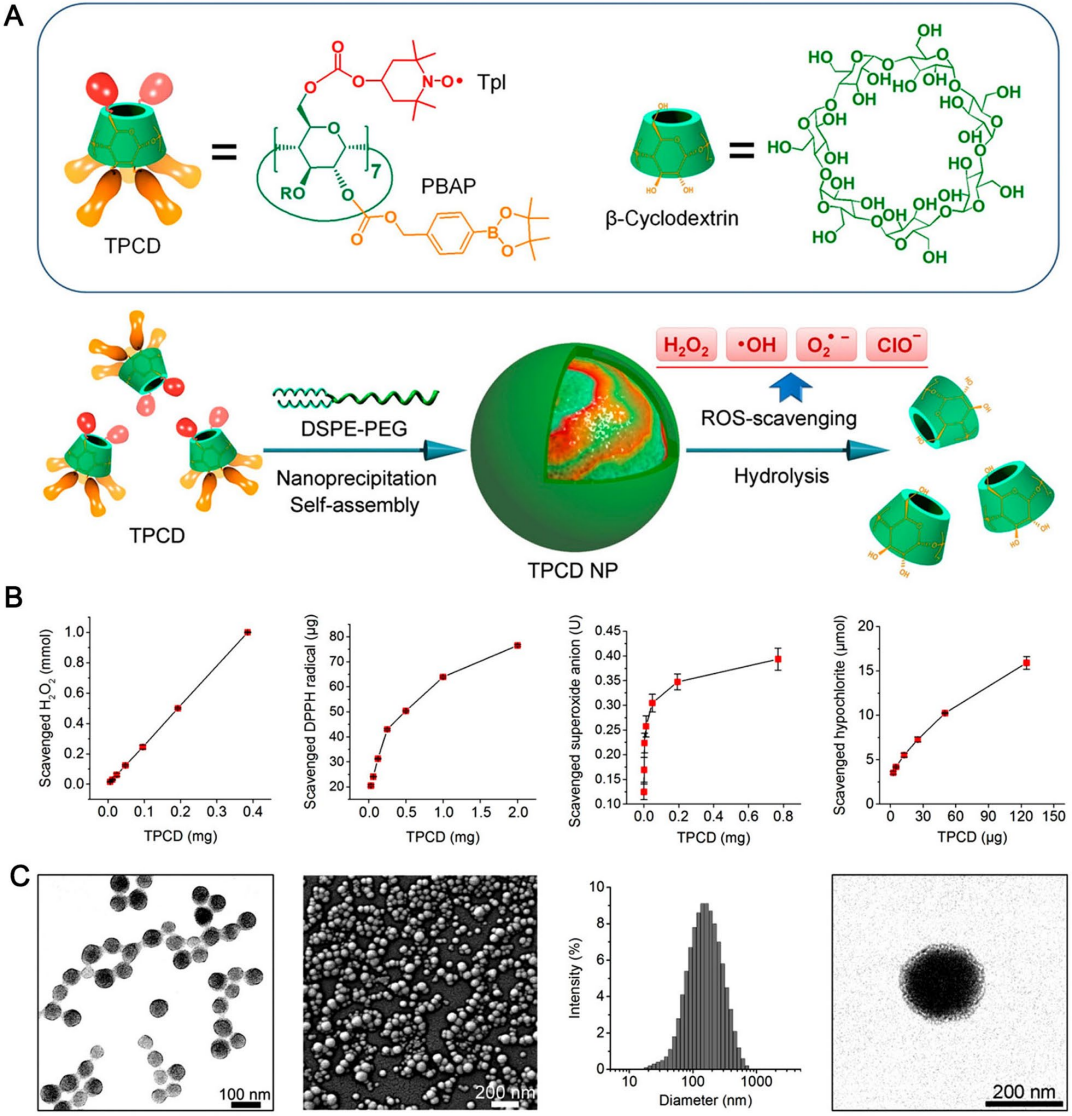
An overview of the design, distribution, and targeting capabilities of a nanoparticle with a wide spectrum ROS scavenging capacity. **A** The creation of a TPCD NP and its chemical structure as a ROS-scavenging substance. **B** TPCD is able to remove H_2_O_2_, DHHP, ·O_2_·, and hypochlorite, with the effectiveness depending on the dosage. **C** Representative transmission electron microscopy (TEM) image, scanning electron microscopy image (SEM), size distribution profile and TEM image after phosphotungstic acid staining of TPCD NPs. Copyright 2018, American Chemical Society

Another study used a SOD-mimetic agent (Tempol) and a H_2_O_2_-eliminating substance of phenylboronic acid pinacol ester covalently conjugated on β-cyclodextrin (β-CD) (TPCD NPs) to treat AS (Fig. 5). TPCD NPs accumulated in atherosclerotic lesions by passive targeting through the dysfunctional endothelium and translocation via inflammatory cells. TPCD NPs reduced systemic and local oxidative stress and inflammation, and eliminated oxidized LDL internalization [145].

Wu et al. covalently bonded Au nanoparticles (Au NPs) to L-Arginine (LA) and β-cyclodextrin (β-CD) to make a NO-driven nanomotor (CD-LA-Au-aV). Modified anti-VascularCellAdhesionMolecule-1 antibody targets and anchored nanomotors to blood vessel walls. LA reduced ROS, β-CD cleared cholesterol in foam cells, and Au NPs killed inflammatory macrophages. Dual-mode nanomotors improved anti-AS efficiency [146].

In order to treat AS, a unique tetrapod needle-like PdH (TN-PdHs) nanozyme [147] that reacted ROS scavenging, anti-inflammation, and autophagy activation was developed. The oxidative alteration of the confined LDL was prevented by the designed TN-PdHs, which also decreased OS in the vessels. They were quite effective in reducing inflammation, as they reduced levels of pro-inflammatory cytokines such as TNF-α, IL-1, and IL-6. Another study prepared a new type of PdH-Tellurium (PdH-Te) nanozyme. This PdH-Te nanozyme not only exhibited intrinsic CAT and SOD-like activities, but also as worked as an excellent H_2_ storage material, both of which can reach effective treatment through a combination of scavenging ROS and anti-inflammation [148].

Using porous manganese-substituted prussian blue (PMPB) nanocubes (NC), Zhang et al. [149] developed a theranostic agent loaded with simvastatin (Sim). The two active components PMPB NC and Sim helped reduce atherosclerotic plaques and inflammation by decreasing ROS levels (free radicals and H_2_O_2_), pro-inflammatory cytokine secretion, collagen accumulation, fibrous cap thickness, macrophage infiltration, foam cell generation, and LDL internalization. Sim as a model drug, Epigallocatechin gallate (EGCG) as an antioxidant agent, and distearyl phosphatidylcholine (DSPC) as major carriers were used to make liposome nanoparticles (SE-LNPs) in the study of Jun Wan et al. [95]. SE-LNPs had a prolonged release profile, allowing the bulk of medication to accumulate at the targeted atherosclerotic plaque, which might resist oxidation, apoptosis, enhance M2 polarization, and decrease blood lipids and lesions. Yue Dai et al. created GPRD NPs by electrostatically adsorbing Gd-doped Prussian blue (GPB), polymer polyethyleneimine (PEI), fluorescent molecule rhodamine (Rd), and targeted molecule dextran sulfate (DS) [150]. GPRD NPs effectively imaged and inhibited AS susceptible plaque in vivo using GPB’s MR and fluorescence imaging, Rd's nano-enzyme, and DS's targeting abilities. GPB NPs had the action without drug loading, simplifying nanocomplex production. Yan Zhu et al. constructed a Prussian blue-based nanomedical loading system with hyaluronic acid (HA) coating, in which colchicine was encapsulated to create col@PBNP@HA [151]. col@PBNP@HA successfully reduced MDA and MPO levels and increased GSH levels, HA on the drug surface specifically bound to CD44 expressed on inflammatory macrophages, which allowed the drug to target plaques to eliminate inflammation.

Jessica Chavez et al. used carbon nanodots (CNDs) in EA.hy926 Endothelial Cells [152]. CNDs effectively scavenged H_2_O_2_ and increased the activity of the antioxidant enzyme NQO1.

Suman Basak et al. drafted novel nitroxide-based nanogels (NGs) crafted through controlled RAFT (Reversible Addition Fragmentation chain Transfer) polymerization to introduce atherosclerosis. Nitroxyl radical-based antioxidants mimic SOD activity, effectively scavenging ROS and reducing LDL oxidation. NGs provided increased surface area, enhanced accessibility of nitroxide groups, higher stability cross-linking, and longer shelf life. NGs effectively reduced foam cell formation and prevents oxidative damage [153].

Many appealing properties of nanoparticles include their tiny size (and consequently huge surface area per volume), relative simplicity of manipulation, and surface components. The survival of nanoparticles in plasma and their permeability in non-targeted organs and tissues must also be explored.

### Age-related pulmonary disease

#### Idiopathic pulmonary fibrosis

Interstitial remodeling is a hallmark of the degenerative lung condition known as idiopathic pulmonary fibrosis (IPF). Telomere shortening, DNA damage response (DDR), and cellular senescence are all linked to pulmonary fibrosis [154, 155]. ROS causes single-stranded DNA damage and breakage, resulting in alveolar epithelial cells (AEC) injury and necrosis via the death receptor route [156], mitochondrial death pathway [157], and endoplasmic reticulum-associated death pathway [158]. Given the compelling evidence connecting OS to the pathophysiology of IPF, targeting ROS may be a successful therapeutic approach.

C60 fullerene has been demonstrated to be capable of scavenging multiple types of free radicals, including ·O_2_·,^1^O_2_, and·OH [159]. At low physiological concentrations, water-soluble C60 is innocuous and possesses significant antioxidant properties. Dong et al. found that water-soluble C60 reduced the severity of bleomycin-induced pulmonary fibrosis in mice [160]. In AEC, water-soluble C60 reduced the concentration of ROS, the expression of TGF-1 and TNF, apoptosis, and/or necrosis. Gadofullerenol (GF-OH m) and fullerenol (C70-OH) NPs were designed as ROS scavengers to inhibit BLM-induced pulmonary fibrosis in a separate study [161]. GF-OH/C70-OH NPs were superior to GF-OH NPs at neutralizing OS and scavenging free radicals.

Yinjuan Lv et al. encapsulated copper-based nanozyme (CuxO NPs) and gold nanoparticles (Au NPs) in oxidation-sensitive dextran (Oxi-Dex) to synthesize ROS-responsive nanocomposites (named as RSNPs) [162]. CuxO NPs showed superior SOD-like and CAT-like activities. RSNPs specifically recognized excess ROS and damaged mesenchymal stem cells (MSCs), released therapeutic nanoenzymes, thereby enhancing the anti-oxidative stress capacity of MSCs and prolonging their survival time in vivo.

Vanadium carbide nanosheets (V_4_C_3_ NSs) were reported to serve as a potential antioxidant for treatment of IPF, which triggers multiple antioxidant mechanisms including electron transfer, H atom transfer, and enzyme-like catalysis [163]. V_4_C_3_ NSs demonstrated significant therapeutic efficacy by scavenging ROS and RNS (ABTS + •, DPPH•, PTIO•,·OH, ·O_2_·, H_2_O_2_), anti-inflammatory activity, and reestablishment of lung antioxidant defenses.

#### Chronic obstructive pulmonary disease

External variables, such as cigarette smoking, air pollution exposure, and occupational exposures, are major contributors to the development of chronic obstructive pulmonary disease (COPD). Increases in oxidative load, ROS and reactive nitrogen intermediates (RNI) [164], which are linked to COPD. COPD patients' neutrophils and airway smooth muscle cells have higher amounts of ROS than those of healthy people [165]. Similarly, neutrophils isolated from COPD patients' peripheral blood have been found to produce higher levels of ROS compared to healthy controls [166]. The degradation of elastin in the lung parenchyma might be hastened by OS, which can disrupt the activity of antiproteases such alpha-1 antitrypsin and secretory leukoprotease inhibitor. OS reduces histone deacetylase activity [167, 168] and boosts histone acetyltransferase activity [169], resulting in increased expression of proinflammatory marks. Both chronic bronchitis and small-airway fibrosis have been linked to OS [170, 171].

Multiple materials have been shown to be effective in treating COPD, with NMs as vectors for enhancing functions. Chitosan (CS) and SLNs were used to encapsulate berberine (Ber) [99]. The effects of Ber pretreatment on MPO and SOD activity in cigarette smoke-induced COPD mice were amplified by Ber encapsulated in SLN-chitosan nanoparticles. The aqueous solubility and oral bioavailability of SLN nanoparticles coated with CS improve the pharmacological effects of Ber. Paudel et al. found that treating human broncho-epithelial cells and macrophages with Ber-loaded liquid crystalline nanoparticles (LCNs) improved its physiochemical properties such as high entrapment efficiency and sustained in vitro release. Ber-LCNs inhibited total cellular ROS, modulated genes associated in inflammation and OS [172].

Likewise, lipopolysaccharide (LPS)-induced oxidative damage in human bronchial epithelial cell line (BEAS-2-B) cells was researched using rutin-loaded liquid crystalline nanoparticles (LCNs). LCNs increased transport, biological activity, treatment regime, and patient compliance. Rutin-loaded LCNs dramatically lowered NO and ROS levels in BEAS-2B cells while also preventing apoptosis [173]. Keshav Raj Paudel et al. evaluated the effect of zerumbone-loaded LCNs (ZER-LCNs) in cigarette smoke extract (CSE)-induced models [174]. The antioxidant activity of ZER is exerted by increasing GSH levels to reduce ROS. ZER-LCN showed greater pharmacological and biological benefits in reducing smoking-induced inflammation, oxidative stress, and aging than free ZER alone.

Dimethyl fumarate (DMF) has antioxidant and anti-inflammatory properties in COPD patients [175]. It reduces OS by activating the nuclear factor (erythroid-derived 2) -like 2 (Nrf2) genetic pathway [176]. Priya Muralidharan et al. [177] created respiratory tract-targeted inhalable DMF dry powders. Solid-state respirable microparticles/nanoparticles dispersed aerosols well, which show the potential to reach lower airways.

Kosuke Chikuma et al. developed a co-delivery approach using core–shell type lipid-polymer nanoparticles (LPNs) with a poly lactic acid (PLA) core carrying a potent antioxidant Mn-porphyrin dimer (MnPD) and a cationic lipid (DOTAP) shell that binds HDAC2-encoding plasmid DNA (pHDAC2). The co-delivery system had low toxicity, high serum stabilities, delayed and tuneable drug release, and excellent drug encapsulation efficiency. PLA-MnPD/DOTAP/pHDAC2 decreased ROS and glucocorticoid resistance in COPD patients [178]. S Castellani et al. used SLNs to encapsulate grape seed extract (GSE) with proanthocyanidins. GSE-loaded SLNs had a longer anti-oxidant impact than free GSE in H441 airway epithelial cells. This formulation may reduce ROS-induced inflammation during chronic lung illnesses [179].

**Fig. 6:**
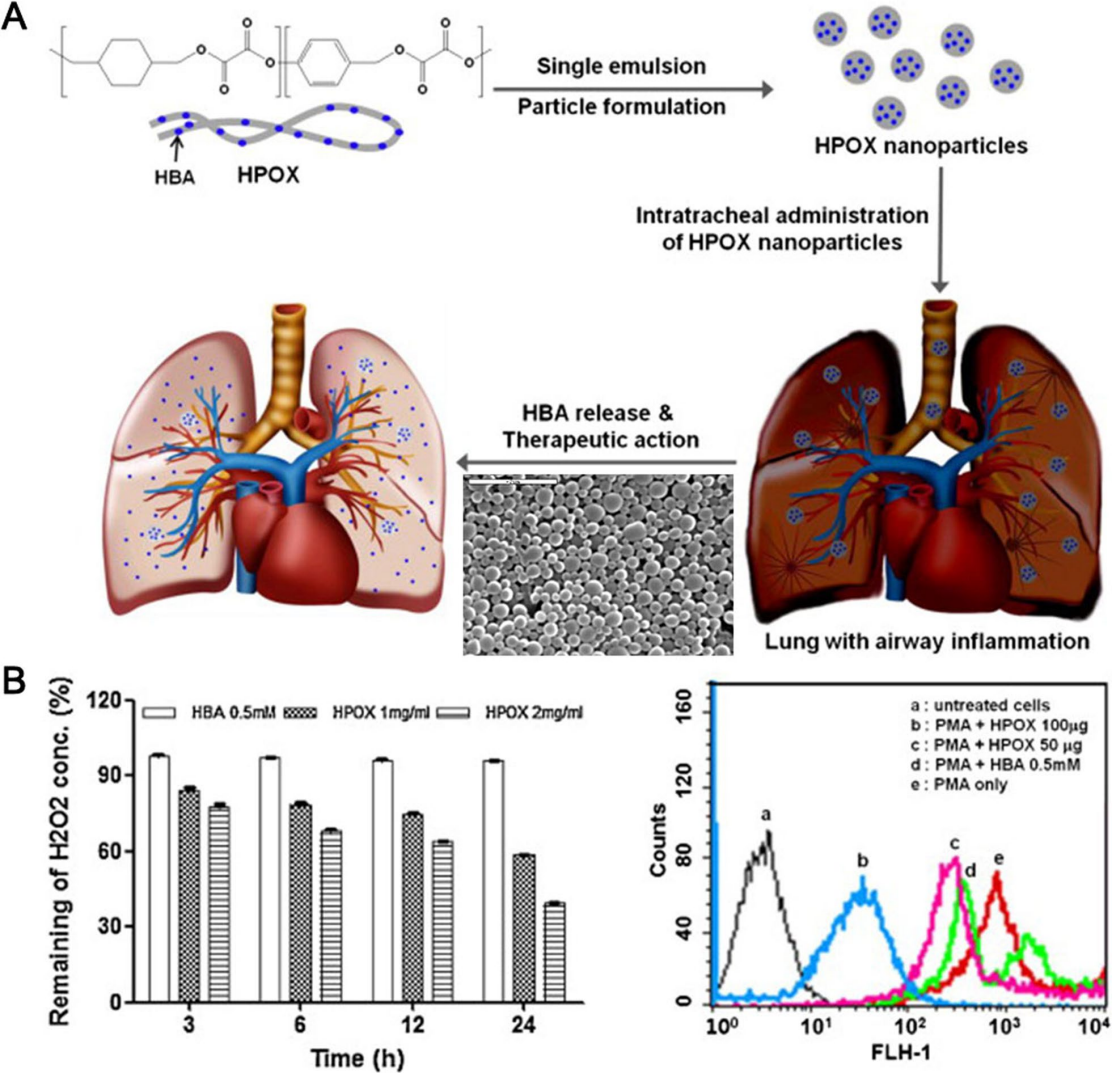
Incorporated polyoxalate (HPOX) nanoparticle structure and antioxidant capacity. **A** HPOX is an innovative prodrug polymer that uses HBA as its backbone antioxidant and anti-inflammatory properties. HPOX’s medicinal actions come from the release of HBA during the breakdown process. **B** The ability of HPOX nanoparticles to scavenge H_2_O_2_ and to suppress the production of ROS in PMA-stimulated macrophages. Copyright 2013, Elsevier

Incorporated polyoxalate (HPOX) may reduce respiratory tract inflammation [180]. HPOX NMs scavenged H_2_O_2_, reduced intracellular OS, and inhibited the expression of pro-inflammatory mediators like iNOS, cyclooxygenase-2 (COX-2), and IL-1β in stimulated macrophages. HPOX NMs were biocompatible and strong antioxidants and anti-inflammatories for airway inflammatory diseases (Fig. 6).

The lungs are unique compared to other systems in that NMs can be administered directly in the lungs to avoid first-pass metabolism, thereby increasing local concentrations in lungs. However, there are still problems such as airway mucus layer barriers, clearance by mucosal ciliary clearance systems, and the need to cross the epithelial barrier for the drug to reach the endothelial cell layer. All these issues need to be considered together in drug design with respect to the chemical-physical properties of the NMs [181]. Currently, research is focused on maximizing delivery efficiency and minimizing toxicity. This includes the PEG-modification on surface and optimization of osmotic pressure gradient for mucus penetration, as well as the optimization of formulation to improve stability, deep lung deposition, and distribution. To successfully transport antioxidants to the lungs, further study is required. The potential for immunogenicity and toxicity to the lungs is an important factor to consider.

### Skeletal and muscle degenerative diseases

**Fig. 7:**
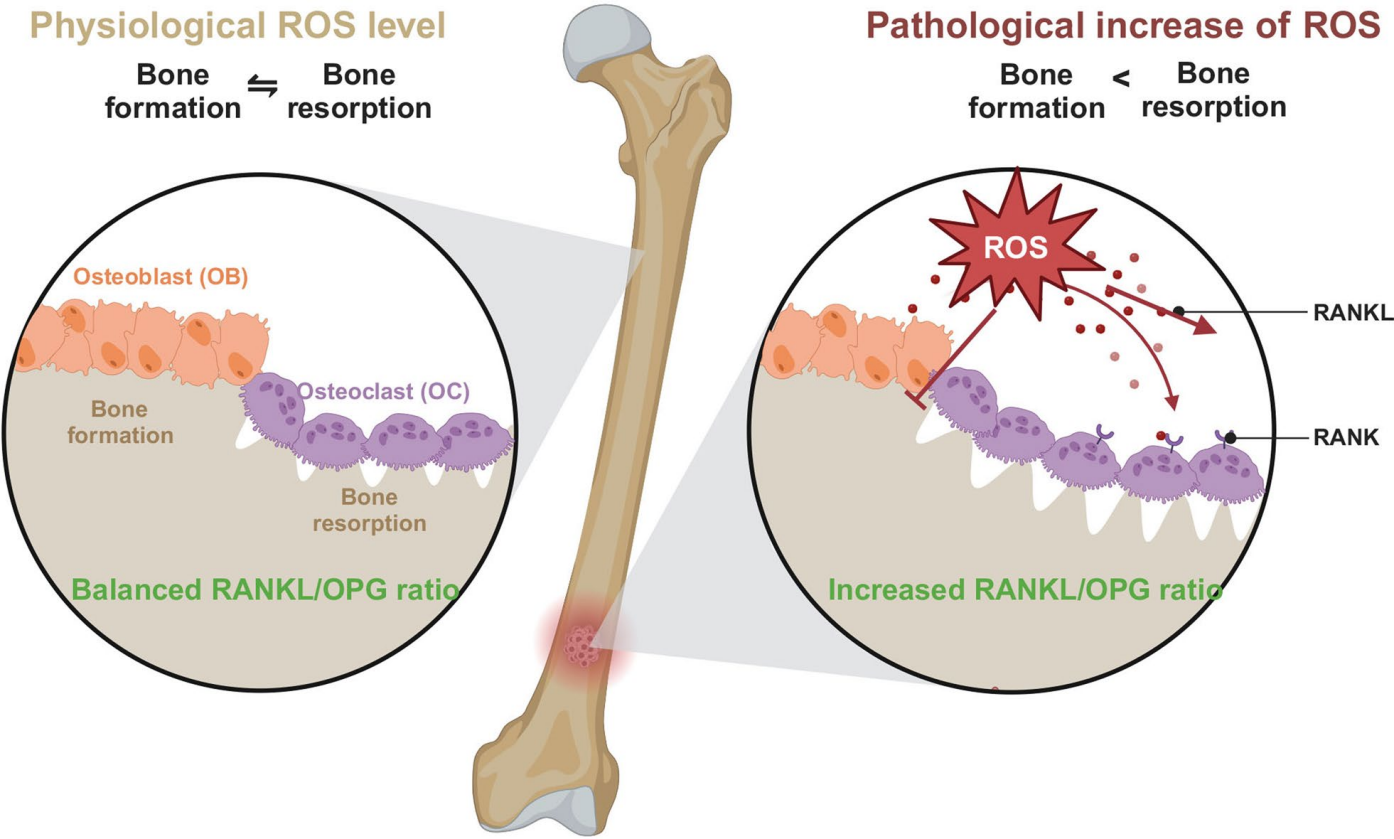
Pathology of skeletal degenerative diseases induced by ROS. Created with BioRender.com

A crucial regulator of osteoclast development, both bone production and bone resorption is receptor activator of nuclear factor Kappa-B ligand (RANKL). Studies have indicated that the osteoprotegerin (OPG), receptor activator of nuclear factor Kappa-B (RANK), and RANKL system may play a crucial role in the process tying osteoporosis and osteoarthritis together (Fig. 7). Interleukin (IL-6, IL-13), TNF, and other inflammatory substances that are released have high osteoclastogenic activity and can either directly activate osteoclast precursors or stimulate RANKL to promote osteoclast formation. Along with the rise in RANKL, a significant amount of RANKL binds to the usual level of OPG, causing a compensatory drop in OPG and an increase in bone resorption [182].

Due to the denser nature of pathological skeletal tissues, high concentrations of drugs are required to achieve efficacy, which can also have toxic effects on other organs. Therefore, the development of well-targeted, highly permeable, slow-release, low-toxicity, and bone-targeted NMs is in the spotlight [183].

#### Osteoporosis

Decreased bone density and degradation of bone tissue microstructure characterize osteoporosis (OP), a systemic and metabolic disease of aging. OP is characterized by increased OC activity relative to OB activity [184]. Patients with OP have a bone microenvironment characterized as immune imbalance and increased OS. Excessive ROS under stressful conditions trigger apoptosis in OBs and osteocytes while encouraging the development and function of OCs [185]. Therefore, enhancing the oxidative state is crucial for osteoporosis therapy and prevention.

Yanhai Xi et al. designed PEGylated hollow gold nanoparticles (HGNPs) loaded with α-Lipoic acid (ALA) (mPEG@HGNPs-ALA) [186]. ALA can suppress intracellular oxidative stress levels and promote the proliferation and differentiation of osteoblasts. In addition to a larger drug loading capacity and enhanced photothermal conversion ability, HGNPs are also tiny (only 30–60 nm in diameter), non-toxic, and spherical in form. The antioxidant capacity and biocompatibility of mPEG@HGNPs-ALA indicated its potential for use in osteoporosis therapy.

The nitrogen-doped carbon dots (N-CDs) have therapeutic promise for the treatment of osteoclast-related osteolytic disorders [187]. The N-CDs decreased Nox1 and upregulated Nrf2 to inhibit RANKL-induced ROS production. Inhibiting osteoclastogenesis and bone resorption with N-CDs in vivo partially protected mice against lipopolysaccharide (LPS)-induced calvarial bone degradation and breast cancer-induced tibial bone destruction. Photoluminescent carbon dots (PCDs) from sour apples cured a mouse calvarial osteolysis model induced by ultra-high molecular weight polyethylene (UHMWPE) wear particles. PCDs reduced UHMWPE-induced ROS stress and pro-inflammatory cytokine production to inhibit osteoclastogenesis and bone resorption in vitro [188].

The osteoporosis cell model examined the ROS-scavenger nanoceria encapsulated in mesoporous silica nanoparticles (Ce@MSNs). Self-regenerating nanoceria mimics SOD and CAT activities. The bioactive MSNs and nanoceria in Ce@MSNs NPs stimulate bone repair and reduce osteoclast activity by releasing osteogenic silica and scavenging ROS. The Ce@MSNs showed promise as a therapy for osteoporosis, based on their potential therapeutic efficacy [189].

The polyglucose-sorbitol-carboxymethyl ether (PSC) was employed as the precursor to synthesize Fe_2_O_3_@PSC NPs in a mouse model of iron accumulation (IA)-related osteoporosis [190]. Nanoscaled Fe_2_O_3_ minimized the generation of free iron ions. PSC protected bone tissues from the damaging effects caused by ROS generation induced by free iron ions. Fe_2_O_3_@PSC sustainably released iron ions instead of releasing a great quantity in a short time, which showed promise as a new IA-related osteoporosis treatment. Iron oxide nanoparticles (IONPs) scavenge ROS through the Nrf2-keap1 pathway to ameliorate postmenopausal bone loss. Zheng et al. created bone targeting IONPs (BTNPs) using alendronate. BTNPs targeted bone surfaces and scavenged ROS to treat mice with ovariectomy-induced osteoporosis. BTNPs outperformed IONPs and bisphosphonates, suggesting a viable clinical use [191].

Polyhydroxyalkanoate-encapsulated CaSi2 nanoparticles (CSN)-loading mesoporous bioactive glass (MBG) scaffolds (CSN@PHA-MBG) were designed for releasing H_2_ in the repair of bone defect of elders [192]. CSN greatly improved H_2_ release capacity for approximately one week. Sustained treatment of H_2_ generally attenuated oxidative stress and effectively remodelled the senescence-associated secretory phenotype via anti-inflammatory pathways, supporting damaged aged bone repair.

Nahida Rasool et al. [193] developed thiolated, bioactive mesoporous silica nanoparticles (MSN-SH) for bone tissue engineering/osteoporosis. Functional modification of the surface thiol groups enhanced the osteogenic properties of MSN and confers antioxidant and cell adhesion properties. MSN-SH neutralized ROS and provide protection against ROS-induced cellular damage.

Conventional therapies have the limitation of side effects and poor penetration into skeletal lesions, while NMs could improve drug solubility and stability [194]. NMs in circulation may still be non-specifically phagocytosed by the liver and spleen, limiting the targeted impact, and this is one of the main reasons why biological NMs are not widely used in the treatment of OP.

#### Osteoarthritis

Osteoarthritis (OA) is a progressive joint disease that is characterized by the deterioration of articular cartilage and oseophyte. OA can affect any joints in the body. Numerous studies point to the role of ROS as primary contributors to the development of OA. The OS caused by ROS is capable of oxidizing cartilage, which will then disturb its homeostasis, encouraging catabolism through the induction of cell death, and harming a variety of components of the joint [195]. ROS operate as inflammatory mediators by activating proteoglycans, collagen molecules, matrix proteins, and membrane proteins directly [196, 197]. These proteins, including IL-1β and TNF-α, are directly responsible for the significant damage that is caused to the joint tissues of OA sufferers. As a result, ROS scavengers have a significant amount of untapped potential for the treatment and remission of OA.

Surface quinone residues in natural melanin efficiently scavenge radicals. Zhong et al. found that dopamine melanin (DM) NPs may scavenge ROS (including ·O_2_·, ·OH) and reactive nitrogen species (RNS), protecting chondrocytes from OS, inflammation, and cartilage degeneration. DM NPs, which were almost 110 nm, may stay in the joint longer than small molecule scavengers, suppressing ROS/RNS and managing OA [198]. MOF-decorated mesoporous polydopamine was utilized by Song et al. to develop a dual-drug delivery system, with rapamycin (Rap) injected into the mesopores and Bi deposited onto the MOF shell. By coupling the collagen II-targeting peptide (WYRGRL) to the nanocarrier, a cartilage-targeting dual-drug delivery nanoplatform (RB@MPMW) was developed. RB@MPMW effectively eliminated cellular ROS through Br and enhances autophagic activity via Rap [199].

The capacity of chitosan nanoparticles with glutathione (Np-GSH) were evaluated in Rats with OA [200]. GSH can directly interact with ROS or act as a cofactor in enzymatic processes. Chitosan-based grafts were ideal substrates for the proliferation of chondrocytes. The GSH contained within nanoparticles (NPs) can be delivered to chondrocytes, reducing ROS, increasing GSH levels and the activity of GPx, and reducing lipid peroxidation.

Haifeng Liang et al. encapsulated melatonin in poly(lactic-co-glycolic acid) (PLGA), with the type II collagen targeting peptide attached to the surface to prepare a nano-delivery system loaded with melatonin(MT@PLGA-COLBP) [201]. Melatonin enhanced the activity of antioxidant enzymes such as GPx and SOD. It repaired the damaged mitochondrial function in chondrocytes and reduces hydroxyl radicals through its metal chelating activity. The MT@PLGA-COLBP formulation achieved targeted functional release and sustained release of melatonin within the joint space, improving cartilage matrix metabolism and delaying the progression of OA in the body.

Exogenous SOD's poor pharmacokinetics and poor cell permeability may explain why native SOD showed no therapeutic benefits. O-HTCC-SOD is a nanoparticle-like compound of cationic functionalized CS and SOD [202]. Due to its highly cationic nanoparticle-like feature, O-HTCC-SOD may penetrate cells and effectively scavenge intracellular ROS. O-HTCC-SOD protected chondrocytes longer than native SOD from monoiodoacetate (MIA)-induced oxidative damage, which included reducing mechanical allodynia, inhibiting gross morphological and histological cartilage lesions, and increasing antioxidant capacity and anti-inflammatory action. Tao et al. used SOD-loaded porous polymersome nanoparticles (SOD-NPs) to target mouse synovium [102]. SOD-NPs had prolonged mouse joint retention time and minimized oxidative damages.

Zhang et al. loaded calcium boride nanosheets (CBN) as H_2_ precursors onto dopamine-modified hydrogel platform (CBN@GelDA hydrogel) for OA treatment. CBN@GelDA hydrogel released H_2_ stably and continuously under physiological conditions, the release process does not affect pH of the microenvironment. CBN@GelDA hydrogel scavenged excessive ROS, alleviated oxidative stress, reduced inflammation and joint destruction, and provided long-lasting relief of OA [203].

Zhao et al. created novel drug-free nanospheres which were self-assembled into spherical aggregates from the block copolymer of P(DMA-*b*-SBMA) in aqueous solution. The nanospheres' clever construction gave them the capacity to withstand physiological stresses, improve lubrication, and neutralize harmful ROS. In a rat model of temporomandibular joint (TMJ) osteoarthritis, the nanospheres prevented structural damage to the condylar cartilage and subchondral bone, slowed the deterioration and ageing of the cartilage matrix [204].

MnO_2_ NPs can function as an artificial nanoenzyme to scavenge ROS. The PEG-MnO_2_ NPs improved chondrocyte viability and extracellular matrix preservation by reducing inflammation-induced OS in cartilage [63]. Chen et al. synthesized an intelligent hollow MnO_2_ (H-MnO_2_) modified with NH_2_-PEG-NH_2_ to target OA treatment [205]. H-MnO_2_ NPs had the ability to efficiently eliminate ROS and greatly alleviate the inflammatory response of OA without evident side effects, opening up new treatment avenues for those living with the condition.

Pei et al. treated OA in rats using water-soluble polyhydroxylated C60 (fullerol) NPs [206]. Fullerol reduced OA by preventing synovial membrane inflammation and chondrocyte destruction in OA joints.

Ruiming Liang et al. suggested using nanofibers constructed of poly (ε-caprolactone) (PCL) and PCL-grafted lignin (PCL-g-lignin) copolymer [207]. PCL tailored mechanical properties whereas ligin had inherent and persistent antioxidant action. Biocompatible, biodegradable, and antioxidant-rich PCL-lignin nanofibrous membranes treated OA.

Compared to traditional spherical cerium dioxide nanoparticles, Urchin-like ceria nanoparticles loaded miR-224-5p more effectively delivered miRNA into cells and exhibit superior ROS scavenging capabilities. This enhanced their ability to suppress inflammatory responses and modulate the microenvironment of OA, thereby improving the gene therapy approaches for OA [208].

Degeneration of the whole joint characterizes OA, making intra-articular injection of ROS-responsive nanomedicine an ideal treatment option, since it allows for regulated release and focused therapy without systemic side effects. Furthermore, NMs should be developed to maximize the retention period in the joint cavity because of the quick clearance of the joint cavity.

#### Sarcopenia

Consistent muscular weakening and atrophy with advancing age was termed sarcopenia [209]. An imbalance between ROS/RNS and the enzymatic antioxidant defence system is a crucial player in the pathophysiological pathways that lead to sarcopenia. Recent studies have shown that compared to young/adult rats, myofibers from elderly rodents contain higher amounts of RONS intracellularly [210]. Muscle mass was negatively impacted by elevated ROS because it facilitated ER stress, which caused cell death in muscle cells. Increased oxidative damage and mitochondrial malfunction, decreased ATP generation, increased protein breakdown, and decreased protein synthesis are all potential outcomes of an overactive redox signaling system in muscle fibers [211, 212].

As a nanocarrier for antioxidants, hydroxyapatite is a material that is often used in sarcopenia. Biocompatibility and biodegradability make hydroxyapatite (HAP) a popular drug delivery system material. The following materials increased curcumin loading surface area. Curcumin-loaded HAP modified with stearic acid (Cur-SHAP) released continuously for over 2 weeks, reducing sarcopenia development or even reversing it [213]. Bletilla striata polysaccharide (BSP) coupled with HAP was employed by Ya-Jyun Liang et al. [213]. BSP is an efficient ROS scavenger. In the current investigation, BSP-HAP administered by intramuscular injection would remain in the muscle tissue, followed by a slow absorption via endocytosis. In the recovery of LPS-induced muscle damage, the created BSP-HAP could decrease LPS-induced ROS formation and improve tissue healing.

Natural antioxidants such as curcumin rather than nanomaterials with ROS scavenging activity are mostly used in the antioxidant treatment of sarcopenia. Nevertheless, the effect of antioxidant supplementation on muscle performance is still highly debatable.

### Skin aging

**Fig. 8:**
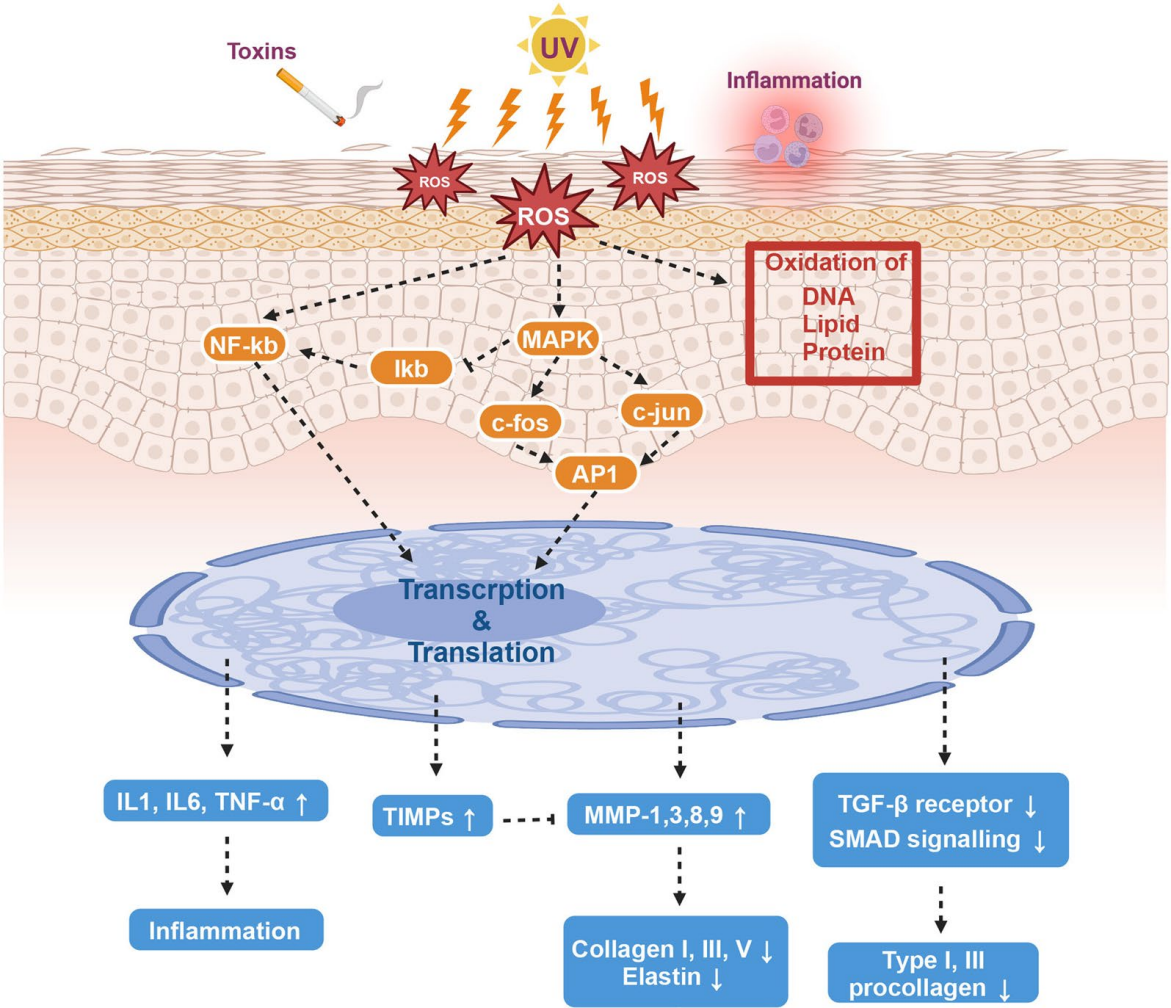
Pathology of skin aging induced by ROS. Created with BioRender.com

Skin aging is characterized by fine lines and wrinkles, loss of elasticity and volume, sagging, roughness and pallor in appearance. The generation of ROS, which causes DNA, protein, and lipid damage as well as extracellular matrix dis-organization, is a typical hallmark of both intrinsic and extrinsic skin aging [214]. Skin has a greater ROS burden when compared to other organs, which impacts both intrinsic and extrinsic aging [215]. Excessive ROS can boost the expression of pro-inflammatory cytokines including IL-1, TNF-α, IL-6, and COX-2 to regulate the inflammatory response [216, 217], as well as make the MMPs/TIMPs ratio imbalanced by activating MMPs and decreasing TIMP production, decompressing ECM [218]. Meanwhile, ROS can suppress collagen formation and accelerate skin aging via regulating the TGF-β/Smad signaling pathway [219] (Fig. 8). Antioxidants have been demonstrated to dramatically reduce or prevent free radical damage to the skin.

Antioxidant nanoparticles have attracted a lot of interest in the cosmetics industry as a possible solution to the effects of skin aging. Investigations on nanoization of conventional medications begun at an early stage, such as EGCG, RSV, CoQ10, quercetin.

Nano-transfersomes loaded with EGCG and hyaluronic acid (HA) were employed by Avadhani et al. [220]. HA's anti-aging qualities, which include biocompatibility, particular viscoelasticity, hydration, and lubrication, make it a promising anti-aging agent [221]. Optimized transfersomes had far greater skin penetration and EGCG deposition than pure EGCG, which improved cell survival, lipid peroxidation, intracellular ROS, and MMP expression in human keratinocyte cell lines (HaCaT).

SLNs and NLCs can provide intimate contact and promote medication absorption via the skin. Incorporating RSV into SLNs and NLCs [96], encapsulating CoQ10 into NLC [100], liposomes (LIPO-Q10) and SLNs (SLN-Q10) [94], ultra-small lipid nanoparticles (usNLC-CoQ10) [222] has been investigated for topical use. All of the above exhibited excellent antioxidant capacity in cells following UVA and UVB irradiation.

A typical dietary flavonoid, quercetin has several physiological benefits including being a powerful antioxidant, scavenger of free radicals, and anti-inflammatory [223]. Tyrosol-incorporated copolyoxalate (TPOX) NPs were synthesized by Kim et al., and they were made up of an H_2_O_2_-sensitive peroxalate ester incorporating tyrosol. Then, quercetin (QTPOX) was included into the TPOX NPs. H_2_O_2_ may delicately break down TPOX into CO_2_ and H_2_O. This sensitive quality helps to target and release in oxidatively damaged cells. In HaCaT cells, the QTPOX NPs demonstrate cytoprotective properties via antioxidative and anti-inflammatory actions [224]. Nisar et al. created Quercetin-loaded zinc oxide nanoparticles (Quercetin@ZnO NPs) in vitro. ZnO NPs release the drug for sunblocking and protecting such as antioxidant, anti-inflammatory, and iron-sequestering properties by delivering maximum quercetin molecules to the targeted site after UVA exposure [225].

The antioxidant capabilities of stable SeNPs stabilized with chitosan of varying molecular weights (Mws) (CS-SeNPs) were investigated. Because of its low toxicity and bioavailability, CS could survive pepsin and pancreatin, and stabilize the Se system in the digestive enzyme environment. In skin-aging mice, all CS-SeNPs penetrated tissues and had antioxidant effects [77].

A fullerene-loaded nanoemulsion was employed to preserve collagen and prevent skin aging [226]. In the HaCaT cell line, Xiao et al. investigated the antioxidant properties of several water-soluble fullerene derivatives. A ROS-scavenging effect against UVB-injuries was demonstrated for PVP/fullerene, CD/fullerene, and hydroxyl group-containing fullerene, indicating the likelihood of skin aging [227].

After UVA radiation, CeO_2_ NPs decreased pro-inflammatory cytokines, intracellular ROS, senescence-associated β-galactosidase activity, and JNK activation [228]. CeO_2_ NPs were used to scavenge ROS, protected skin against radiation and inflammation, and helped wounded healing [229, 230].

Enzyme-mimicking Au-Pt nanocomposites (NCs) were produced by Xiong et al. in HaCaT cell lines to scavenge cellular ROS caused by UV irradiation [231].

Chiral manganese dioxide nanoparticles with high sensitivity and selectivity for ROS were engineered. MnO_2_ NPs eliminated ROS in skin tissues, increased collagen, and showed exciting roles in inhibiting oxidative damage in skin and preventing skin aging [232].

Redox nanoparticles (RNP^N^) are nitroxide radical-containing polymers that may efficiently remove ROS. Oral RNP^N^ supplementation increased the therapeutic benefits of the core nitroxide radical and decreased UVB-induced skin aging in an inflammatory skin model. The RNP^N^ may protect skin against ROS damage and slow aging [233].

Nanotechnology can improve the performance of cosmetics in a variety of ways, such as by enhancing entrapment efficacy, physical stability and dermal penetration of the active ingredient, regulating the release of the active ingredient. The majority of these bioactive compounds, however, are poorly absorbed by the skin. On the one hand, the skin permeability of nanomedicines needs to be enhanced, and on the other hand, nanoparticles may cross the skin and enter the body circulation, causing unintended toxicity and side effects. Nanoparticles may cause skin irritation or allergic reactions. It is necessary to adjust the size, shape, charge, degradability, and dose of the drugs to make them more absorbable, less toxic, and less allergenic [234].

### Neurodegenerative diseases

**Fig. 9:**
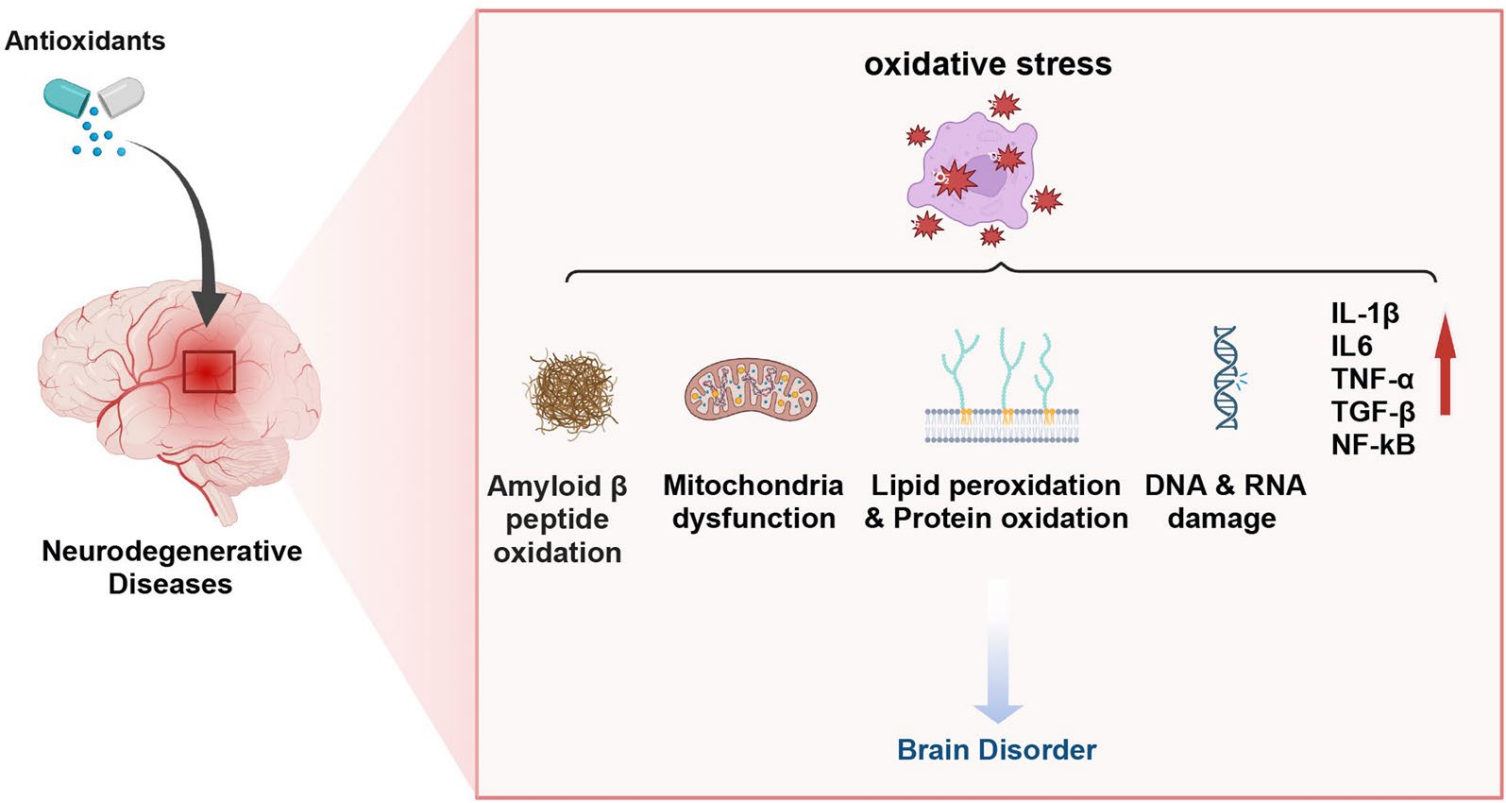
Pathology of neurodegenerative diseases induced by ROS. Created with BioRender.com

Alzheimer's disease and Parkinson’s disease are the two most prevalent neurodegenerative diseases, respectively. In terms of mechanisms of OS, there are many commonalities between AD and PD. ROS production played an important role in Amyloid-beta (Aβ) oxidation, mitochondrial dysfunction, upregulation of inflammatory factor expression and selective neuronal degeneration (Fig. 9).

The existence of the blood–brain barrier (BBB) hinders the identification and treatment of brain illnesses by limiting the transit of biologically active chemicals and drugs [235]. Drugs were unable to sustain a high enough bioavailability to have an impact on the brain parenchyma pharmacologically. The unique qualities of NMs, including as their tiny size, drug-loading capacity, high blood stability, low immunogenicity, high biodegradability, and the ability to change surface properties, have been employed to treat neurodegenerative illnesses [236].

Although NMs can effectively penetrate the BBB to reach the brain, they may accumulate in the brain and thus cause adverse reactions or toxicity. In addition, the long-term stability and metabolic pathways of nanoparticles in the body are not yet fully understood, and there may be a risk of long-term accumulation and chronic toxicity. NMs may activate the immune system of the human body, triggering an inflammatory response and affecting the health of the nervous system [237, 238].

Translated with DeepL.com (free version).

#### Alzheimer's disease

AD is a neurodegenerative disease characterized by gradual cognitive decline and behavioral abnormalities, with common clinical symptoms including progressive forgetfulness, loss of recognition, loss of reading, and loss of speaking. Neurodegenerative illnesses like AD are characterized by OS and neuronal death [239]. Accumulation of free radical caused damage and altered expression of antioxidant enzymes are hallmarks of AD [240]. Due to reduced production of major antioxidant enzymes as CAT, SOD, GPx, and GSH reductase, the body is unable to utilize its detoxification mechanisms effectively [241]. Together, the oxidative imbalance, the overexpression of nuclear factor kappa-light-chain-enhancer (NF-kB), and the release of inflammatory mediators (such as IL-1β, IL-6, TNF-α, and TGF-β) create an environment conducive to the development of AD [242, 243]. The activation of N-methyl-D-aspartate receptors (NMDARs) results in ROS production when Aβ accumulates abnormally. This in turn led to OS. ROS triggers a vicious loop that causes the onset and development of AD by increasing the synthesis and aggregation of Aβ and hyperphosphorylated Tau. Antioxidant treatments have emerged as promising possibilities for treating AD, according to preclinical research [244]. Additionally, several types of nanoparticles have been utilized in AD investigations (Table 3).

**Table 3:** Application of ROS-scavenging nanomaterials in treatment of Alzheimer's disease

Type	Drug	Nanocarrier/modification	Cell and animal model	Mechanism	References
Nanomaterials with ROS scavenging activity	nitroxyl radical-containing nanoparticles (RNPs)		human neuroblastoma SH-SY5Y cells	Decrease ·O_2_· and ·OH	[245]
	MeO-PEG-b-PMNT redox nanoparticle [RNPN]		17-week-old senescence-accelerated prone (SAMP8) mice	Increase SOD activity, reduce OS, improve neuron survival and cognitive improvement	[246]
	Se-chondroitin sulfate nanoparticles (CS@Se)		human neuroblastoma SH-SY5Y cells	Decrease the levels of ROS and MDA, enhance GPx	[247]
	borneol (Bor)-modified octahedral Pd (Pd@PEG@Bor) nanozyme platform		SH-SY5Y cells, 3 × Tg-AD mice	Reduce ROS and Ca^2+^ contents, keep the mitochondrial membrane potential stable, reduce Aβ plaque formation, neuronal death, and neuro-inflammation	[248]
	Resveratrol-Se-Peptide Nanocomposites(TGN-Res@SeNPs)		PC12 cells, d-gal-induced AD model mice	Increase CAT, and GPx and total antioxidant capacity, normalize MDA levels, decrease Aβ aggregation, down-regulate Aβ-induced neuroinflammation, alleviate gut microbiota disorder	[249]
	ROS-responsive dendrimer-peptide conjugate (APBP)		SH-SY5Y cells, APPswe/PSEN1dE9 model mice	Reduce ROS, decrease Aβ burden, minimize glial cell activation, improve cognitive abilities	[250]
	Reduced polyoxometalates (rPOMs)	mesoporous silica nanoparticles (MSNs)	PC12 cells, wild-type mice	prevent Aβ aggregation, reduce ROS, and create local hyperthermia to disaggregate Aβ fibrils	[251]
	Au NPs	mesoporous silica	PC12 cells	Decrease the Aβ self-assembly, inhibit Cu^2+^-induced Aβ40 aggregation and diminish Aβ40-Cu^2+^ complex-induced membrane rupture, microtubular abnormalities, and ROS-mediated death	[252]
			AD model rats generated by intracerebroventricular–streptozotocin	Increase SOD and CAT, prevent mitochondrial ATP production, neuroinflammation and OS	[253]
		maize tetrapeptide-anchored Au NPs	PC12 cells, AD mice induced by aluminum chloride and d-galactose	Inhibit intracellular ROS accumulation and promote cell differentiation, enhance the functionality of neuroprotection	[254]
	Iron oxide nanoparticles		Drosophelia	Mimic CAT and breakdown ROS, inhibit apoptosis, increase mobility	[255]
	CeO_2_ NPs		SH-SY5Y cells	Decline Aβ1-42 aggregation, protect against neurotoxicity of ROS generated by Cu^2+^ + Aβ1-42, block free radical formation and scavenge radicals	[256]
			5XFAD transgenic AD model mice	Prevent neuronal death by scavenging mitochondrial ROS and lowering OS, gliosis, and mitochondrial damage	[66]
		integrating antioxidative nanozymes (ceria) into MOF	SH-SY5Y cells	Demonstrate strong SOD and CAT mimetic actions that eliminate ROS and prevent its oxidative damage to newborn neurons, increase their survival rate and outgrowth	[257]
		gold nanorods (Au NRs)	PC12 cells, APP/PS1 mice	Mimic CAT and SOD, enhance the BBB permeability, extend the photocatalytic activity of CeO_2_ to the near-infrared (NIR) region	[258]
	PB NPs	polyamidoamine (PAMAM) dendrimer/Angiopep-2 (PPA) nanoparticles	BV-2 microglia cells, APP/PS1 model mice	Mimic CAT, SOD, POX, display higher BBB permeability and synergistically scavenge ROS and restore microglia mitochondrial activity, diminish neurotoxic Aβ aggregation	[259]
		modification with transferrin and Congo red	PC12 cells, APP/PS1 mice	Scavenge ROS, alleviate cognitive decline, hippocampus atrophy, and AD-related pathology	[260]
Nanomaterials as carriers	Resveratrol	Lipid-core nanocapsules	Rats received intra-cerebroventricular injection of Aβ1-42	Decrease intracellular OS, caspase-3 activity and cytotoxicity prevent Aβ-induced behavioral deficits, astrocyte and microglial activation, and cell signaling disruptions	[261]
		Polymeric micelles	PC12 cells	Suppress mitochondrial ROS, scavenge ROS, and inactivate caspase3 to protect numerous cell types from Aβ toxicity	[262]
		red blood cell (RBC) membrane-coated nanostructured lipid carriers	APP/PS1 mice	Penetrate BBB, target neuron cells, localize to mitochondria, ameliorate memory impairment	[263]
	Curcumin	PLGA NPs	APP/PS1 mice, HT22 cells	Increase the APP/PS1 mice's capacity for spatial learning and memory, decrease hippocampus-amyloid formation and deposit, and diminish tau hyperphosphorylation	[264]
		PLGA NPs	Aβ-induced AD rat model	Inhibit H_2_O_2_-induced Nrf2 activation, enhance neuronal differentiation, stimulate adult neurogenesis	[265]
		PLGA NPs	SK-N-SH cells	Inhibit H_2_O_2_-induced ROS and GSH consumption	[266]
		PBCA NPs	SH-SY5Y cells	Have antioxidant and antiamyloidogenic properties, enhance curcumin absorption and drug release	[113]
		Polymeric nanomicelles		Reduce amyloidogenesis by glycation and limit the development and accumulation of amyloid fibrils, nullifying free radicals' effect	[114]
		NLCs	AD rats generated by injecting Aβ into hippocampus	Decrease hippocampus tissue ROS production, lipid peroxidation, and ADP/ATP ratio	[267]
		NIPAAM/VP/AA polymeric nanoparticle	SK-N-SH cells, athymic mice	Increase GSH levels and reduce H_2_O_2_ and caspase 3 and 7 activity in the brain	[268]
		Fe3O4@carbon dots nanocomposite	PC12 cells	Suppress extracellular Aβ fibrillation, ROS generation mediated by Aβ fibrils, and neurotoxicity, and have a high affinity for Aβ	[101]
	quercetin	magnetic core–shell mesoporous silica nano-formulations	neuronal hippocampal cells	Reduce of Aβ cellular toxicity, prevent Aβ fibril-mediated ROS generation and neurotoxicity	[244]
	silibilin	human serum albumin (HSA) nanoparticles	SH-SY5Y cells	Recover cell viability, increase SOD, CAT, and GSH content, decrease ROS, Caspase-3 activity and fragmentation of DNA	[269]
	Anthocyanins	PLGA@PEG nanoparticles	SH-SY5Y cells	Reduce the OS caused by Aβ1–42 and increase the expression of Nrf-2 and HO-1 proteins	[111]
	Phytol	PLGA nanoparticles	Wistar rat scopolamine model of AD	Activate antioxidative defense system (SOD and CAT), replenish GPx, and control apoptotic cell death	[112]
	α-bisabolol	solid lipid nanoparticles	Neuro2A cells	Suppress the production of ROS/RNS, protect the cells from Aβ induced apoptosis	[270]
	vitamin E	polyethylene glycol-based nanospheres	SH-SY5Y cells	Prevent Aβ-induced ROS after Aβ exposure	[106]
	tocopheryl polyethylene glycol succinate-1000	multi-functional PAMAM	SH-SY5Y cells	Reduce the ROS activity, reduce Aβ1-42-induced apoptosis, show mitigation of Aβ1-42-induced toxicity in neuronal cells	[271]
	ferulic acid	Solid lipid nanoparticles	LAN5 human neuroblastoma cells	Decrease ROS production, restore mitochondrial function, activate of the intrinsic pathway of apoptosis	[97]
	carotenoids	cationic biopolymer core/shell nanoparticles (Chitosan@PLGA C/SNPs)	SH-SY5Y cells, wild type male Sprague Dawley (SD) rats	Increase CAT activity, reduce MDA level, aid in intranasal Lutein delivery to the brain	[103]
	methyl gallate	starch nanoparticles	Neuro2A cells	Mitigate ROS-mediated macromolecular damage, restore mitochondrial membrane potential and attenuate apoptosis, attenuate aggregation of Aβ peptide and disaggregate the preformed amyloid plaques	[272]

Due to site-specific delivery, the ability to cross the BBB, increased drug solubility, and greater therapeutic efficacy, nano-delivery is a preferable alternative. BBB penetration favors particles with a lower dimension. It is essential to use nanoparticles of a reduced size to improve BBB penetration, reduce acute toxicity and adverse effects, and increase drug loading capacity.

#### Parkinson’s disease

Parkinson's disease (PD) is a degenerative disorder of the central nervous system that slows the mobility of the patient. The early manifestations of the disease include resting tremor, myotonia, slow movement, difficulty in starting movements and abnormal posture. In PD patients, Farias et al. [273]discovered elevated lipid hydroperoxides (LOOH), MDA levels, and SOD activity, as well as reduced CAT activity. ROS-mediated OS is closely related to PD, mainly because the production of large amounts of ROS by activated microglia is accompanied by increased sensitivity to ROS and reduced scavenging capacity of brain tissue in PD patients [274]. Additional pathways involved in PD are neurodegeneration caused by the action of androgen receptors [275], enhanced α-synuclein aggregation and formation of oxidatively modified forms of α-synuclein [276], degradation of quinone oxidoreductase 1 [277], attenuation of protein DJ-1’s deglycase activity [278], activation of gene LRRK2 [279], decreased tetrahydrobiopterin and tyrosine hydroxylase (TH) metabolism [280]. Numerous new pharmaceutical therapeutics targeting the OS pathway have been developed, and they are proved useful in the treatment of PD. Here, we discuss the use of ROS-scavenging nanotechnology for PD therapy (Table 4).

**Table 4:** Application of nanomaterials ROS-scavenging nanomaterials in treatment of Parkinson’s disease

Type	Drug	Nanocarrier/modification	Cell and animal model	Meachnism	References
Nanomaterials with ROS scavenging activity	CuxO nanoparticle clusters (NCs)		SH-SY5Y cells, 1-methyl-4-phenyl-1,2,3,6-tetrahydropyridine (MPTP) induced mice	Have the activities of POD, SOD, CAT, and GPx, inhibit neurotoxicity and rescued the memory loss	[282]
	nanochelating based nano complexes		PC12 cells	Improve cell survival, SOD and CAT activity, reduce Caspase 3 expression, and inhibit methyl-4-phenylpyridinium (MPP( +))-induced ROS and mitochondrial membrane potential loss	[283]
	Nitroxide Radical-Containing Redox Nanoparticles (NRNPs)		SH-SY5Y cells	Decrease superoxide levels, protect neuronal cells against the 6-OHDA-induced damage	[284]
	V2O5 NPs		HEK293T cells, HeLa cells, LNCaP cells, SH-SY5Y cells	Display robust GPx mimetic activity, exhibit significant cytoprotective effects against OS	[285]
	PtCu nanoalloys (NAs)		primary cortical neurons, α-syn preformed fibrils -induced mice	Inhibit OS, cell-to-cell transmission, and neurotoxicity by scavenging ROS, along with POD, CAT, and SOD-like activities	[286]
	tris malonic acid C60 adducts (carboxyfullerenes)		cortical astrocytes	Eliminate ·O_2_· and H_2_O_2_, halt the loss of mesencephalic dopaminergic neurons caused by MPP( +) and 6-hydroxydopamine (6-OHDA)	[287]
			MPTP primate model of PD	Reduce ROS associated with neurodegeneration, increase parkinsonian motor scores, striatal fluorodopa and dihydrotetrabenazine uptake, and striatal dopamine concentrations	[288]
	Superparamagnetic Iron Oxide Nanoparticles (SPIONs)		6-OHDA rat model of PD	Improve mitochondrial dysfunction and resistance to OS	[289]
	CeO_2_ NPs		yeast cells with heterologous expression of the human α-syn	Inhibit cytoplasmic α-syn foci accumulation, reduce ROS and α-syn-induced mitochondrial dysfunction	[290]
			SH-SY5Y cells, MPTP-induced mice	Scavenge ROS, inhibit the microglial activation and lipid peroxidation, while protecting the TH	[291]
			MPTP-induced mice	Act as free radical scavengers, elevate striatal dopamine level and improve motor performance	[292]
			6-OHDA induced rats	Preserve striatal dopamine and protect dopaminergic neurons in the substantia nigra, antioxidant and antiapoptotic effects	[293]
		Yb^3+^, Er^3+^ Codoped	MPTP-induced PD mice	Promote the activities of GPx and total antioxidant capacity increase, exhibit biocompatibility and antioxidant catalytic properties	[294]
	Mn3O4 NPs		SHSY-5Y cells, MN9D cells/ PD mice	Have SOD, CAT and GPx activity, scavenges ·OH; reduce α-syn in PD rats' CSF, enhance cognitive function, and biodegrade in vivo	[295, 296]
Nanomaterials as carriers	CoQ10	lipid	Wild type rats	Show high bioavailability and more anti-lipid peroxidation activity	[297]
	CAT	Exosomes	PC12 cells, 6-OHDA model mice	Accumulate in neurons and microglial cells in the brain, deactivate ROS and protective nerves	[298]
		PLGA NPs	primary human neurons	Reduce H_2_O_2_-induced protein and DNA damage, mitochondrial membrane transition pore opening and membrane damage, restore neuronal normal function and microtubule-associated protein-2 levels	[110]
	Resveratrol	polysorbate 80-coated poly(lactide) nanoparticles	MPTP-induced model mice	Attenuate MPTP-induced lipid peroxidation neurotrophic and anti-apoptotic efficacy	[299]
		Liposomal formulation	DJ-1-gene knockout rat model of PD	Increase levels of GSH and SOD, decrease the level of MDA, inhibit apoptosis and reduce motor impairment	[300]
	Curcumin	lactoferrin nano particles	SK-N-SH cells	Decrease ROS, anti-apoptotic and neurotrophic effects	[301]
	Nicotine and caffeine		MPTP-induced parkinsonism mice	Ameliorate the increase in lipid peroxidation, show greater dopaminergic neuron endurance, fibre expansion, and TH and growth-associated protein-43 expression against MPP( +)-induced changes in vitro	[302]
			MPTP-induced parkinsonism mice	Eliminate multiple ROS such as H_2_O_2_, ·OH, and ·O_2_·, increase bioavailability, improv neuroprotection	[109]
	quercetin	cell membrane coated novel biomimetic Cu2-xSe-PVP-Qe nanoparticles	SH-SY5Y cells, MPTP-induced model mice	Target microglia, display multienzyme activities scavenging ROS, polarize microglia into the anti-inflammatory M2-like phenotype to reduce neuroinflammation	[303]

Nanomaterials have great antioxidant qualities, however their applications raise certain safety concerns. In the neurological system, NMs may cause apoptosis, release ROS, modify the production of pro-inflammatory cytokines, and affect neurotransmitter expression [281]. Protecting the brain's homeostasis against the effects of nanoparticles and their breakdown products is an urgent need.

### Reproductive aging

#### Female reproductive aging

The adult hypothalamic-pituitary system, also known as the hypothalamic-pituitary-ovarian (HPO) axis, coordinates with the follicles in the ovaries to control menstrual cycles and the reproductive lifetime and healthspan. With increasing age, follicles are gradually depleted and their quality declines, leading to reproductive aging and menopause. This process is reflected in a significant age-related increase in the probability of infertility, miscarriage and birth defects in the offspring [304]. ROS is considered to be responsible for the initiation or development of pathological processes affecting ovarian function [305]. Follicle atresia and decreased oocyte quality and quantity may result from excessive ROS, which damage DNA, disturb protein function and homeostasis, promote ER stress, autophagy, and proteasome dysfunction among other detrimental effects [306]. Pathological ROS drive ovarian aging by apoptosis, mitochondrial dysfunction, inflammation, telomere shortening and other aspects [307–309]. Related antioxidants, such as MLT, vitamin E, and resveratrol, could improve ovarian function and therefore have potential clinical applications [310, 311]. Unfortunately, there is a dearth of studies on the impact of nano-antioxidants on ovarian aging.

#### Male reproductive aging

Decrease in sperm quality and a higher chance of birth abnormalities and disorders in progeny are signs of reproductive aging in males [304]. 15% of couples worldwide struggle with male infertility, making it an important health issue that has to be addressed head-on [312]. According to recent research, 25–40% of infertile men have high ROS levels in their semen [313, 314]. The integrity of sperm DNA is similarly compromised by OS, which may have an impact on the ability of embryos to grow and the health of their progeny. Male reproductive potential may be decreased by age-related OS because of deteriorating semen quality, altered endocrine, and sexual dysfunction [315]. Patients with elevated levels of ROS may benefit from antioxidant treatment [316], and it is important to design the reasonable antioxidants for male reproductive aging.

The great majority of antioxidants in male reproductive aging are nanoparticles with their own ROS scavenging activity, and some of these are also being utilized in conjunction with traditional medicines like MLT to maximize their capabilities.

MLT is a powerful antioxidant that is capable of capturing free radicals. Synthesized gold (III) MLT (Au^3+^/MLT) complexes showed anti-inflammatory and antioxidant properties to protect against testicular injury [317]. MLT is an effective formulation for scavenging ROS, triggering the production of molecules that protect sperm from oxidative stress. The combination of Au + 3/MLT significantly enhances total antioxidant capacity compared to using MLT alone.

By reducing OS, Nanoform Se (NSe) reduced testicular toxicity and apoptosis cause by BPA or NiSO_4_ [318, 319]. NSe was more protective than Se [319].

(FSH)-conjugated SOD-loaded PLGA NPs designed by Snow-Lisy et al. targeted testis Sertoli cells to combat male infertility caused by high levels of ROS [320].

Ionizing radiation produces ROS through the radiolysis of water in irradiated testicular tissue, which causes spermatogenic cell mutation or death, reduced sperm quantity and motility, and increased sperm deformity rate. Molecular hydrogen (H_2_) has the potential to be a radioprotective agent due to its ability to scavenge ·OH selectively. The use of MgH_2_ nanoparticles for hydrogen storage and release have several benefits, including high storage capacity, a smooth release rate, and great stability. Ma et al. [321] observed that MgH_2_ reduced MDA levels in testis, inhibited ROS formation after irradiation, and removed ·OH. Furthermore, by neutralizing hydroxyl free radicals, MgH_2_ therapy enhanced male fertility impairment due to irradiation.

Ce NPs' potential protective impact against fipronil-induced testis damage was investigated in a rat model [322]. The Ce NPs mitigated the deleterious effects of fipronil on testicular tissue by reducing lipid peroxidation, apoptosis, inflammation, and boosting antioxidant activity.

Fullerenol C60(OH)_24_, a hydroxylated derivative of fullerene, is investigated for its NO-scavenging action in mesenchymal cells from rat testicles in a separate research by Mirkov et al. [323]. C60(OH)_24_ could scavenge ·O_2_· in xanthine/xanthine oxidase system.

There was promising evidence that antioxidants might slow the aging of the male reproductive system. However, an imbalance between oxidants and antioxidants, known as reductive stress (RS), can be caused by an overabundance of antioxidants. The fertility rate and three fundamental seminal indicators (motility, concentration, and morphology) have all been linked to RS's negative consequences [324]. The fertilization process was decreased owing to the inhibition of significant functional activities of the spermatozoa [325]. Therefore, precision antioxidant may be the way forward for study into the effects of aging on reproduction.

### Ocular neurodegenerative disease

#### Age-related macular degeneration

Age-related macular degeneration (AMD) is a chronic neurodegenerative and progressive disease with a multifactorial aetiology that leads to alterations in the macula region's structure [326]. Non-neovascular (‘‘dry’’) AMD effects approximately 85–90% of patients, whereas neovascular (‘‘wet’’) AMD affects the residual 10–15% of patients. Due to its high oxygen metabolism requirements, high unsaturated fatty acid content, the presence of photosensitive molecules (retinoids and lipofuscin), and protracted exposure to light, the retina is more susceptible to injury induced by ROS and OS [327]. Oxidative damage is a precursor to the development of AMD and is implicated in AMD-related inflammation and neovascularization. Key to secondary oxidative injury in the retina [328] are disturbances in the regulation of OS-related molecular pathways such as autophagy and Nrf2 signaling pathways. Given the importance of OS in the pathogenesis of AMD, excessive ROS-targeting antioxidant therapies have been proposed as the first-line treatment.

In order to better administer medications like polydopamine and lutein, nanomaterials are modified to have an enhanced dosage form and permeability. Jiang et al. produced anti-angiogenetic protein-loaded polydopamine NPs for wet AMD [329]. Polydopamine NPs reduced angiogenic agent expression by scavenging ROS stimulated by external OS. In reaction to OS, the particles controllably released loaded anti-angiogenic medicines to cure wet AMD. Lutein is commonly used as an antioxidant due to its ability to quench singlet oxygen and eliminate ROS [330]. However, lutein's inadequate water solubility limits its absorption and effectiveness. Ying Ge et al. created a penetratin-modified lutein nanoemulsion in-situ gel (P-NE GEL) to cure rat dry AMD produced by NaIO_3_. GEL solution significantly extends the corneal retention time of drugs. With the aid of penetratin, P-NE is rapidly transported to the posterior segment of the eye and distributed in the retinal area. P-NE GEL strongly inhibited cell apoptosis and ROS in human retinal epithelial cells (ARPE-19), indicating its potential use in AMD therapy [104]. By modulating Nrf2 via the PI3K/AKT/mTOR signaling pathway, astragaloside-IV (ASIV) may reduce OS. Three different sized ASIV lipid nanocapsules (ASIV-LNCs), sized at 20, 50, and 90 nm, were loaded with a phospholipid complex produced from ASIV [331]. LNCs offer reduced toxicity, increased drug loading capacity, and enhanced permeability. In a mouse model of dry AMD caused by NaIO_3_, the ultra-small-size LNCs (ASIV-LNCs-20) exhibited superior penetration effects, which were able to lower ROS generation and the rate of cell death.

**Fig. 10:**
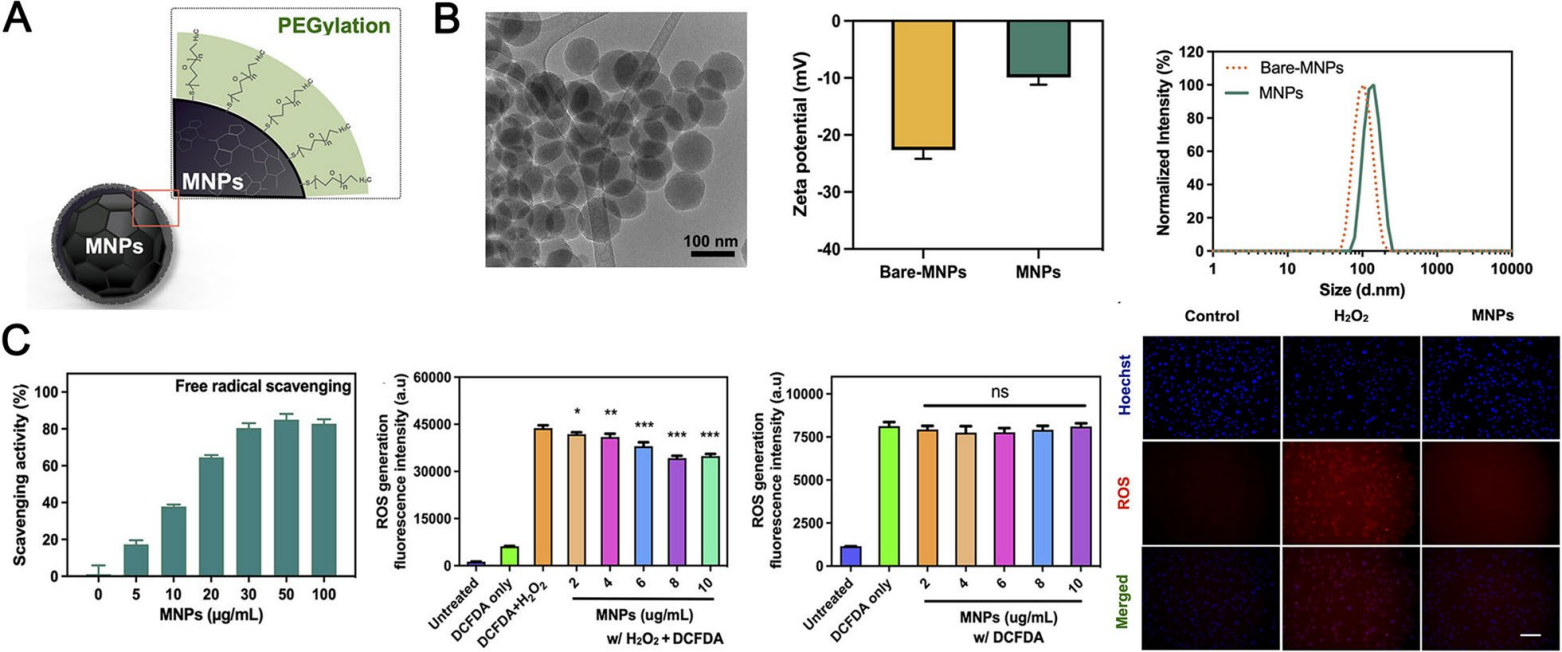
Illustrations and characterizations of MNP schematics. **A** Schematic of the MNP synthesis and characterization. **B** TEM, hydrodynamic dimensions, and Zeta-potential of Bare-MNPs and MNPs. **C** MNPs’ ROS-scavenging activity in ARPE19 cells. Copyright 2022, American Chemical Society

Due to its tiny particle size, NMs with free radical scavenging action offers a distinct advantage in ocular illnesses. Mitra et al. developed water-soluble, biocompatible, trackable nanoceria formulation glycolchitosan-coated ceria nanoparticles (GCCNPs) with enhanced antioxidant ability to scavenge intracellular ROS. In laser-induced AMD, GCCNPs decreased ROS-induced pro-angiogenic vascular endothelial growth factor (VEGF) expression, cumulative oxidative damage, and endothelial precursor cell recruitment without toxicity [332]. Fullerenol (Fol) decreased ROS, normalized GPx activity, and promoted CAT in H_2_O_2_-induced RPE senescence [333]. Its nanosize permitted intravitreal injection into the retina and RPE cells. Yong-Su Kwon et al. [334] used PEGylated synthetic melanin-like nanoparticles (MNPs) in the RPE to restore melanin for AMD therapy. Biocompatible MNPs preferentially targeted ROS with significant antioxidant effects. MNPs could also treat AMD pathology with a single treatment (Fig. 10).

Absorption rates, medication penetration, active solubility, and bioavailability have all been proven to enhance with the use of nanomedicine. The absence of blood flow at the location of sickness is common in the eye since it is a relatively closed organ. The creation of a nanocarrier for topical use in the eye is urgently required. When designing a dosage, it's important to keep nanomaterial complexity and dose to a minimal. The clinical translation of methods for sustained and targeted administration of nanoscale medicines to the posterior portion of the eye to treat AMD is still an area of active research.

#### Cataract

Cataract is primarily an ARD, with a loss of transparency in the lens of the eye, manifesting as blurred vision or glare. The buildup of primary lipid peroxidation (LPO) products (diene conjugates, cetodienes) was characteristic of the early stages of cataract. The preponderance of end LPO luminous products was characteristic of the later stages [335]. Cataractogenesis has been linked to ROS that cause damage in the lens cell, which may take the form of protein oxidation, DNA damage, and/or lipid peroxidation [336]. Antioxidants are a potentially effective strategy for managing cataracts as well as a variety of ocular disorders of the aging eye caused by ROS.

Ethylene glycol, ethylene glycol monoacetate, and ethylene glycol diacetate (EGCNPs) coated cerium oxide nanoparticles were produced in a work by Hanafy et al. Elevated level of reduced GSH to oxidized GSH (GSH/GSSG) in human lens epithelial cells (HLECs) was a result of EGCNPs displacing POD activity [337].

### Renal aging

The aging process is associated with a variety of structural and functional changes in the kidneys and a decreased ability to recover from a kidney injury, both of which contribute to long-term renal outcomes: over 60 percent of people aged 80 and older are diagnosed with chronic kidney disease (CKD) [338, 339]. The loss of renal mass, glomerulosclerosis, glomerular basement membrane hypertrophy, tubular atrophy, interstitial fibrosis, and arteriosclerosis are associated with aging kidneys [340, 341]. Renal aging and CKD are linked to elevated OS levels [342]. Multiple studies have linked an increase in ROS markers to a decline in kidney function beginning in the early phases of CKD in adults and children [343, 344]. As the disease progresses, OS indicators such as mitochondrial superoxide, oxidized LDL, homocysteine, SOD, and GSH deficiencies increase in concentration [345, 346]. This overall increased oxidative burden may contribute to chronic cellular stress, mitochondrial injury, apoptosis, and may induce tubular cell injury [347, 348]. Antioxidants are potential anti-aging strategies for the kidneys.

Yuh-Feng Lin et al. attached anti-kidney injury molecule-1 antibodies to resveratrol-loaded PLGA nanoparticles (KIM-1-Res NPs). The unique KIM-1-Res NPs may accurately control medication release, directly target damaged kidney cells, limit side effects, and improve therapeutic results. Molecular-1-Res NPs decreased creatinine and prevented tubulointerstitial damage in CKD mice [349].

In order to protect renal cells from oxidative damage, Fong-Yu Cheng et al. [350] investigated whether thapsigargin (TG)-encapsulated PLGA nanoparticles (TG-PLGA NPs) might promote autophagy. Nrf2 and forkhead box, class O (FoxO1) were activated by the TG NPs to rescue HK-2 cells from OS-induced cell death. Through the production of ER stress and its downstream pathways, the antibody-conjugated TG NPs reduced kidney dysfunction and damage.

As a result of its ability to shield thiol-containing proteins (antioxidant enzymes), zinc has been touted as a pro-antioxidant agent [351]. In order to tackle CKD [352], researchers employed a combination of spironolactone (SPL) and zinc oxide nanoparticles (ZnO-NPs). The antioxidant and anti-inflammatory effects of ZnO-NPs significantly improved the therapeutic efficacy of SPL in the treatment of CKD.

Although ROS-scavenging nanoparticles have promising anti-aging effects, they may potentially trigger OS and mitochondrial dysfunction in the kidneys if used in excess. Several fundings have shown that multi-walled carbon nanotubes (MWCNTs) [353], AuNPs [354], Silver nanoparticles (AgNPs) [355], copper nanoparticles (CuNPs) [356], Pt NPs [357] could induce renal injury. The trade-off between biological toxicity and therapeutic efficacy of nanoparticles remains to be explored in more depth in future studies. NMs may accumulate in the kidneys due to their small size and unique surface properties. The extent of bioaccumulation due to repeated dosage over long periods of time is still unknown [358] [359].

## Clinical trials of ROS-scavenging nanotechnology in treatment of ARD

Even though ROS-scavenging nanotechnology has been the subject for the treatment of ARD, only a handful of these treatments have advanced to the stage of clinical trials (Table 5). The current clinical trial studies about ROS-scavenging nanotechnology suffer from scarcity of trial conduct, small sample size, heterogeneity of study population, diversity of antioxidants, and absence of uniform clinical endpoint indicators. Further studies comparing ROS-scavenging nanotechnology with traditional antioxidants or combinations of them are even more scarce. The efficacy and safety of many antioxidants are currently unknown. and more research, especially clinical trials, are needed to further validate them. The creation, translation, clinical studies, and even the drive toward actual patient usage of nanotechnology still have a great deal of unfinished business.

**Table 5:** Clinical Trials of ROS-Scavenging Nanotechnology in treatment of ARD

Nanomaterials	Disease	NCT Number	Phase	Results	Patient number	References
The novel bioavailable Curcumin (Cureit)	Sarcopenia	CTRI/2018/05/014176	Not available	Cureit is effective in the treatment of sarcopenia due to its anti-inflammatory properties, enhanced hand grip strength, antifatigue properties, and muscle protein control	30	[360]
Nanoparticle Gel from Phyllanthus amarus (PP)	Osteoarthritis	AMSEC-60EX-019	Not available	The anti-inflammatory and pain-relieving properties of PP may help with knee discomfort	30	[361]
Curcumin nanomicelle	Asthenoteratospermia	IRCT2016072519669N2	2–3	Asthenoteratospermia treatment with curcumin nanomicelles may enhance sperm quality	60	[362]
Oligosaccharide nanomedicine of alginate sodium (ONAS)	Osteoporosis	Not available	Not available	ONAS improved complication rates, fusion rates, and Japanese Orthopaedic Association ratings	96	[363]
hyper-harmonized fullerene water complex	Skin aging	Not available	Not available	Products enabled faster regeneration of collagen and prompt skin reaction to the negative environmental influences	38	[364]
nano-curcumin	T2DM	IRCT20130811014330N4	3	Nano-curcumin might prevent AS progression and subsequent cardiovascular events in diabetic cardiac patients	64	[365]

## Conclusions and future perspective

In this review, we provided an introduction to ROS-scavenging nanomaterials, discussed their use in the study of aging, and outlined directions for future research. There are significant obstacles to the clinical translation of ROS-scavenging nanotechnology in aging and ARD, despite the encouraging results from preclinical investigations and clinical trials. Nanomaterials that can scavenge ROS have the potential to outperform current antioxidant treatments, increasing human longevity and enhancing quality of life. However, there are still issues to be resolved, such as the effectiveness of nanoparticles for targeted delivery, the safety of nanomaterials, and a dynamic monitoring system for antioxidant nanomedicine. The root cause, location, lesion micro-environment, and gene expression/signaling pathway modifications of each illness are unique. Nanomaterials should be developed with these features in mind.

Overall, current enhancements in nanomedicine primarily focus on:

*Precision targeting* The development of nanomedicines with targeted capabilities ensures the concentration of drugs at lesion sites, thereby reducing the impact on healthy tissue. This strategy significantly enhances treatment precision. Existing antioxidant-based treatments lack specificity for dysfunctional cells, tissues, and organelles. Antioxidants are frequently not designed to act selectively on senescent cells, which creates uncertainty regarding their actual efficacy and biosafety. In addition, certain biological barriers can impede the accumulation of nanomaterials at disease sites and reduce the efficacy of therapies. Nanodrug delivery may be severely hampered by the non-specific absorption of nanodrugs by healthy organs, one of the common biological barriers. Several strategies have been proposed to combat non-specific absorption by extending the half-life of nanodrugs in circulation. Clinical contexts have utilized PEGylated NP strategies that inhibit clearance by the reticuloendothelial system (RES) or mononuclear phagocyte system (MPS) [102, 111]. Advancements in nanoparticle surface functionalization, such as pH, redox, and light responsiveness. Targeting ligands is also emerging as a promising avenue of research. Nanodrug surface modification of targeting ligands can identify overexpressed receptors in pathological tissues and facilitate site-specific nanodrug delivery [366]. Many targeting ligands such as aptamers, nanoantibodies, small molecules and peptides [367], have been widely used for tumor-targeting nanodrugs. While non-tumor disorders are the principal indication for ROS-eliminating nanodrugs, identifying appropriate ligands will be a fruitful field of study. Drug distribution is greatly hampered by the BBB, a critical barrier in a number of neurological illnesses. The BBB has been approached using a variety of approaches, including chemical alteration of medicines and prodrugs, local distribution mediated by NPs, disruption of the BBB, and different nanocarriers that can cross the BBB [212, 368].

*Biodegradable materials* There’s a concentrated effort to use natural, biocompatible, and easily biodegradable materials for creating nanoparticles. This approach aims to minimize toxicity and the risk of bioaccumulation, ensuring that these nanoparticles can be safely decomposed and cleared from the body post drug release.

*Optimized nanoparticle design* Fine-tuning the size, charge, shape, solubility, and surface properties of nanoparticles can improve their distribution and excretion in targeted tissues, reducing systemic toxicity. For example, smaller nanoparticles in the reproductive system have been linked to reduced sperm count and vitality, potentially leading to damage in cumulus cells and hindering egg maturation [369]. It's observed that cationic nanoparticles typically exhibit greater toxicity than their neutral or anionic counterparts. Surface modifications of nanoparticles, such as glycosylation, acetylation, PEGylation, or peptide modification, can enhance biocompatibility, decreasing immune responses and toxicity. Furthermore, the synergistic use of adjuvants, like permeation enhancers in the skin, can temporarily alter the skin's barrier function to boost the transdermal absorption of nanomedicine. For instance, in cardiovascular applications, stimulus-responsive nanoparticles that react to changes within the blood vessels (such as shear stress) or to external stimuli, like magnetic and temperature-sensitive nanoparticles, present innovative therapeutic possibilities [370].

*Controlled release systems* High reactivity, poor storage ability, and limited bioavailability during in vivo distribution characterize antioxidants. Because encapsulation techniques rely mostly on the passive release or diffusion of antioxidant chemicals, they can't be used for sustained and regulated treatment. Antioxidants in nanomaterials that escape before they reach the site of action may have diminished therapeutic efficacy or even harmful side effects. Enhanced hydrophobic contacts, electrostatic interactions, van der Waals forces, π—π stacking, hydrogen bonding, and covalent bonding are only some of the common interactions used to stabilize nanomaterials for drug delivery platforms [371, 372]. Due to its removal in an acidic intercellular environment, MnO_2_ and ZnO could be utilized as gatekeepers to efficiently restrict medication leakage. The release of antioxidants may need to be balanced with the biodegradability of the biomaterial. Many loaded antioxidant components are released too quickly, in an incomplete form, or are unstable after release [373]. Nanotechnology-based controlled release systems represent a future-worthy area of development, which enable precise drug delivery, improved bioavailability, targeted therapy with minimal side effects, and the capability for simultaneous multi-drug delivery.

*Safety assessment* There is also worry over the toxicity caused by ROS that are created by nanomaterials [374]. In particular, metal nanoparticles can affect the expression of neurotransmitters [281], trigger inflammatory responses, and cause OS. Small nanoparticles generate more ROS [375], because they have a larger specific surface area and greater surface reactivity than larger nanomaterials. Nanomaterials' ability to generate ROS is influenced by a number of physical and chemical characteristics. Too much ROS can be produced if the Fenton reaction speeds up (as it could if the concentration of Cu^2+^ and Cu^+^ were both raised). By generating oxygen radicals and causing the oxidation and cross-linking of protein thiol groups necessary for cell viability, Se in excess leads to apoptosis [376]. Intensify research into the safety of nanomedicines, encompassing systematic evaluations of their biodistribution, metabolic pathways, long-term stability, and potential toxicity within the body.

The majority of antioxidant clinical trials have been conducted on patients with established pathology. However, once senescent cells manifest, antioxidants are unable to reverse their condition. In our summary of above studies, nowadays research concentrate more on synchronous intervention or post-modelling treatment. Prevention of disease may be more realistic than cure. On the individual oxidative status, the interaction of multiple compounds from diet or supplements, the optimal type of antioxidant, exact dosage, treatment intervals, and total duration of therapy, there are numerous unanswered questions. ROS-based nanomaterials should be combined with other therapeutic methods for improved outcomes. The production or elimination of intracellular ROS is dynamic in space and time when nanomaterials are introduced [377]. The development of techniques to monitor and identify the capabilities of particular ROS in real time is also crucial and essential. It’s best to keep things as straightforward as possible when designing nanomaterials, as more intricate structural and functional designs make manufacturing in bulk more challenging and less reliable.

## Data Availability

Data will be made available on request.
